# Socioeconomic position and the impact of increasing availability of lower energy meals vs. menu energy labelling on food choice: two randomized controlled trials in a virtual fast-food restaurant

**DOI:** 10.1186/s12966-020-0922-2

**Published:** 2020-01-31

**Authors:** Lucile Marty, Andrew Jones, Eric Robinson

**Affiliations:** Department of Psychological Sciences, University of Liverpool, Eleanor Rathbone Building, Bedford Street South, Liverpool, L69 7ZA UK

**Keywords:** Food choice, Energy labelling, Availability, Socioeconomic position, Executive function

## Abstract

**Background:**

Food consumed outside of the home is often high in energy and population level interventions that reduce energy intake of people from both lower and higher socioeconomic position (SEP) are needed. There is a lack of evidence on the effectiveness and SEP equity of structural-based (e.g. increasing availability of lower energy options) and information provision (e.g. menu energy labelling) interventions on food choice.

**Methods:**

Across two online experiments, participants of lower and higher SEP made meal choices in a novel virtual fast-food restaurant. To be eligible to take part, participants were required to be UK residents, aged 18 or above, fluent in English, have access to a computer with an internet connection and have no dietary restrictions. Participants were randomized to one of four conditions in a 2 × 2 between-subjects design: menu energy labelling present vs. absent and increased availability of lower energy options (75% of menu options lower energy) vs. baseline availability (25% of menu options lower energy). Participants also completed measures of executive function and food choice motives.

**Results:**

The analysis of pooled data from both studies (*n* = 1743) showed that increasing the availability of lower energy options resulted in participants ordering meals with significantly less energy on average (− 71 kcal, *p* <  0.001, partial η^2^ = 0.024) and this effect was observed irrespective of participant SEP. Menu labelling had no significant effect on energy ordered (− 18 kcal, *p* = 0.116, partial η^2^ = 0.001) in participants from both higher and lower SEP. Furthermore, we found no evidence that executive function or food choice motives moderated the effect of increasing lower energy menu options or energy labelling on total energy ordered.

**Conclusions:**

In a virtual fast-food environment, energy labelling was ineffective in reducing total energy ordered for both higher and lower SEP participants. Increasing the availability of lower energy options had an equitable effect, reducing total energy ordered in participants from higher and lower SEP.

**Trial registration:**

Study protocols and analysis plans were pre-registered on the Open Science Framework (https://osf.io/ajcr6/).

## Background

Increases in population level energy intake have been identified as a key contributor to the development and maintenance of the worldwide overweight and obesity problem [1–3]. Although a number of factors explain why large numbers of people are consuming more energy than is optimal for health, eating out of the home (OOH) may play an important role. In the UK, more than one quarter (27%) of adults and one fifth (19%) of children eat meals out once per week or more [4]. The frequency by which people eat out of the home is associated with greater daily energy intake and overweight/obesity [5–7]. This relationship may be explained by the excessive energy content of the meals in both full service and fast- food restaurants [8]. Although full service restaurants offer significantly more excessively calorific main meals compared with fast-food restaurants, fast-food consumption has been linked to higher caloric intake and greater risk for obesity [9, 10]. In addition, people of lower socioeconomic position (SEP) might be particularly at risk because deprived areas have an increased density of fast-food outlets in the UK [11]. In the context of the overweight and obesity crisis and social inequalities in diet and health, these findings underline the need to identify effective interventions that can reduce the amount of energy (kcal) ordered and consumed in the OOH food sector and specifically in fast-food settings [12–14].

A commonly used intervention approach involves providing the general public with nutritional information about food in order to motivate healthier food choices (‘information provision interventions’). An example of information provision is energy labelling on restaurant menus; a public health policy now implemented federally in the USA [15] and regionally in Ontario, Canada [16] and in New South Wales, Australia [17]. Although energy labelling may have a positive impact on diet through a variety of channels (e.g., reformulation of menus), encouraging customers to choose healthier meals is one of its central purposes [18]. The best available evidence on the effect that energy labelling has on meal choices in the OOH food sector showed a small reduction of energy purchased per meal (− 7.8%, 95% CI: − 13.1% to − 2.5%) [19]. A different intervention approach is to change the structural properties of the food environment in which individuals make food choices (‘structural-based interventions’). In the context of eating out, this could involve reducing meal portion sizes [20] or increasing the proportion of meals on restaurant menus that are lower in energy content. To date, relatively few studies have investigated the impact of altering the availability of food products on energy selected, but a recent systematic review showed a substantial reduction in mean energy selected (− 35.6%, 95% CI: − 59.9% to − 11.7%) [21]. A key difference between information provision and structural-based interventions is the level of individual agency required [22]. Because information provision interventions require conscious motivation and effort on the part of the individual, whereas structural-based interventions do not, it has been argued that structural-based interventions are likely to be more effective than information provision interventions [23]. However, there have been few direct comparisons of the effectiveness of information provision vs. structural-based interventions on food choice, particularly in the context of the OOH food sector. In the present studies, we directly compare the effect of an information provision (energy labelling) vs. a structural-based intervention (increasing availability of lower energy options) on simulated food choice.

Information provision interventions require engagement of a set of cognitive abilities called ‘executive functioning’ [24]; individuals need to attend to information, hold that information in mind and then consciously act on that information. In addition, for provision of nutrition information to impact on behaviour, individuals also presumably need to be motivated by health when making food choices [25, 26]. Because lower education levels – a measure of socioeconomic position (SEP) – have been shown to be associated with reduced executive functioning [27, 28] and being less motivated by health when making food choices [29, 30], it has been argued that information provision interventions may be less effective in lower SEP population than in higher SEP [22, 31]. Conversely, because structural-based interventions do not rely on cognitive abilities or motivation, they may be more equitable and benefit all, irrespective of SEP [32]. Yet, there has been limited testing of the equity of population level interventions to improve nutritional quality of diet. For example, recent reviews have noted that it is unclear whether the effectiveness of menu energy labelling and the impact of altering the availability of healthy vs. less healthy food options differ based on SEP [19, 21, 33].

In the present studies, we made use of an online virtual fast-food environment to study the impact of an information provision and a structural-based intervention on hypothetical food ordering. Online virtual environments allow for recruitment of large and diverse samples to examine simulated food choice under tightly controlled experimental conditions. Online environments have been used to study the effect of a range of intervention types in the context of supermarket shopping behaviour [34, 35], but here we made use of a newly developed online virtual fast-food restaurant to examine simulated food choice. We conducted two studies using the same basic methodology; the second study was planned to address any methodological concerns arising from the first study and to examine replicability of findings. The main aim of the present studies was to compare the effect of energy labelling vs. increased availability of lower energy options on food choice in lower and higher SEP individuals. We also aimed to explore whether SEP differences in executive functioning or food choice motives may explain why these interventions may be more effective for some people than others (e.g. higher vs. lower SEP). We hypothesised that increasing the availability of lower energy options would have a significant effect on total energy ordered, and this effect would be observed irrespective of participant SEP [21]. In line with recent evidence, we hypothesised that menu energy labelling may have a modest effect on energy ordered [19] and this effect would be primarily observed among higher SEP participants. We also hypothesized that if SEP moderated the effect of energy labelling on energy ordered, it may be explained by higher SEP participants having better executive functioning and/or being more motivated by health when making food choices.

## Methods

### Data collection

Two randomized, controlled, pre-registered online experiments were conducted using the same design. Participants were recruited through the platform Prolific Academic between May and August 2019. Participants were eligible to participate if they were UK residents, aged of 18 or above, fluent in English, had access to a computer with an internet connection and had no dietary restrictions. Participants from Study 1 were ineligible to participate in Study 2. In addition, any participants who failed a quality control question (i.e., an attention check) were screened out of the study and their data was not analysed (see Additional file 1: Section 1). We intended to recruit a sample stratified by gender (approx. 50/50), highest educational qualification (approx. 60% A-level or below, 40% above A-level) and student status (approx. 3.5% of students, in Study 2) to be broadly representative of the adult population in England [36, 37]. Eligible participants who completed the study received monetary compensation in return for their participation. The two experiments were approved by the Health and Life Sciences Research Ethics Committee at the University of Liverpool (reference: 4612). Informed consent was obtained from all the participants.

### Study design

In a 2 × 2 between-subjects design, participants from Study 1 and Study 2 were randomly allocated to one of the following conditions: ‘baseline availability’ and ‘no energy labelling’ (C), ‘baseline availability’ and ‘energy labelling’ (CL), ‘increased availability of lower energy options’ and ‘no energy labelling’ (A), ‘increased availability of lower energy options’ and ‘energy labelling’ (AL). Randomisation with 1:1:1:1 allocation was performed using the ‘Random class’ Microsoft algorithm [38].

### Measures of SEP

#### Education level

Our primary measure of SEP was education level because it most closely captures the opportunity to develop knowledge and skills that may affect executive functioning and make a person more receptive to health information [39] and higher education level is associated with use of nutrition information when eating out [40]. We collected two similar, but distinct measures of education level; highest educational qualification and total years in higher education. Highest educational qualification was measured using the question “What is your highest educational qualification?” See Additional file 1**: Section 2** for full response options. A level or below qualifications (high-school completion or below equivalent for US) were categorised as ‘lower education level’ whereas qualifications above A level (above high school equivalent for US) were categorised as ‘higher education level’, as in Best & Papies, 2019 [41]. Years in higher education was measured using the question “After leaving school (i.e. at 16 years old), how many further years of higher education (i.e. a formal course) did you study for?” To account for both the level of qualification achieved and time spent in education, we calculated a continuous composite score (‘level of education’) of the z-scores for highest educational level and years in higher education.

#### Other SEP measures

Participants were asked to report the annual after tax income of their household including all earners to the nearest £1000. Equivalised household income [42] was calculated by dividing the after tax household income by the sum of the equivalence value of all the household members (first adult = 1, additional adult or child aged 14 and over = 0.5, child aged 0–13 = 0.3). To measure perceived SEP participants rated where they think they stood in society from 1 (people who have the least money, least education and the worst jobs or no job) to 10 (people who have the most money, most education and the best jobs) using the MacArthur scale of subjective social status (SSS) [43].

### Additional demographic measures

Age, gender, ethnic group, student status, height, weight, dieting status and fast-food consumption frequency were recorded. Self-reported body mass index (BMI) was calculated in kg/m^2^.

### Virtual fast food restaurant ordering task

#### Virtual fast-food restaurant

**Fig. 1:**
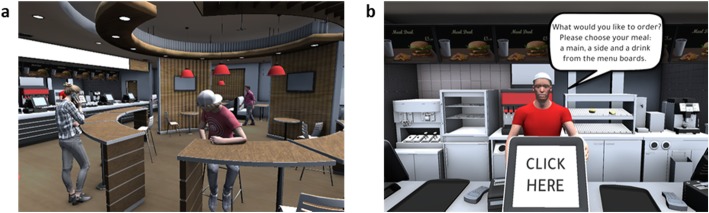
Virtual fast-food restaurant developed on Unity Software. **a** Inside of the virtual fast food restaurant. **b** Virtual fast food restaurant checkout

The virtual fast-food restaurant was designed using Unity [44] and modelled on a popular fast-food chain in the UK (Fig. 1a). The participants clicked on a door to enter the restaurant and navigated around the interior of the restaurant using mouse clicks. The interior included other diners and an ambient background noise of a fast-food restaurant. Once at the counter, the participants were asked by the cashier what they would like to order and clicked on menu boards to make their choice (Fig. 1b).

#### Menu boards

The menu boards included a picture and a name for each food item, based on 2019 menu options of a popular fast-food chain in the UK. In addition, the more detailed description of each food item appeared on the screen when the user moved the cursor over the food item. The number of food items on the menu boards (Study 1: 8 mains, 4 sides and 4 drinks; Study 2: 12 mains, 7 sides, 7 drinks) was chosen to be representative of the number of options advertised on in-store menu boards. The energy content of food and drink items was taken from the website of the fast-food chain. As there is no widely accepted definition of higher vs. lower energy for fast-food products, for the purpose of the present study food items were categorised as ‘lower energy’ (LE) vs. ‘higher energy’ (HE) based on the distribution of energy content of products being sold by the fast-food chain: LE mains ≤400 kcal, HE mains ≥500 kcal, LE sides ≤100 kcal, HE sides ≥200 kcal, LE drinks ≤10 kcal, HE drinks ≥40 kcal.

In the ‘baseline availability’ conditions (C and CL), the proportion of LE vs. HE meal options was similar to the proportion of lower vs. higher energy options in UK fast-food chains i.e. 25% LE vs. 75% HE in each category (mains, sides, drinks) [8]. In the ‘increased availability of lower energy options’ conditions (A and AL) the proportion of LE and HE food items was reversed, i.e. 75% LE vs. 25% HE. The number of food items in each category and the food items with the highest and the lowest energy content in each category were the same across availability conditions. In the ‘energy labelling’ conditions (L and AL) energy in kcal was added on the menu boards for each item and reference information on kcal requirements was clearly displayed at the bottom of the menu: “On average women need 2,000 kcal per day and men need 2,500 kcal per day” [45]. In the ‘no energy labelling’ conditions (C and A) no kcal information was included. To control for price across conditions, a fixed meal price based on an average price of a meal in the fast-food chain (£5.09) for all meal combinations was presented. Images of the menu boards for mains are presented in Additional file 1: Section 3 – Figure S1 & Figure S2 and menu items for each condition are described in Additional file 1: Section 3 – Table S1 & Table S2.

#### Primary outcome: total energy ordered

Participants were asked to choose a main, a side and a drink by clicking on the food item they wanted to order from each menu board. The primary outcome was the total energy ordered from the virtual fast-food restaurant in kcal (including selected main, side and drink).

### Health motives

To assess participants’ health motives in their food choices, we used the single-item Food Choice Questionnaire [46] that includes 11 items about food choice motives. Of relevance to the present study, participants rated the following statements “It is important to me that the food I eat on a typical day: 1/ is healthy (healthiness motivation); 2/ helps me control my weight (weight control motivation)” on a scale from 1 (Not at all important) and 7 (Very important).

### Executive functioning

#### Inhibition

Inhibition is the ability to suppress impulsive or automatic responses (e.g., not choosing unhealthy foods). A Stroop task was used to measure inhibition because performance on this task has been previously related to poverty and excess energy intake [47, 48]. We implemented the classic Stroop task with keyboard input on Inquisit software [49]. Participants saw colour words written in colour and were asked to indicate the colour of the word by key press as fast as they could without making too many errors. The task included congruent trials where colour word and the colour it was presented in were the same, incongruent trials where colour word and the colour it was presented in were not the same, and control trials with coloured rectangles. The task included four colours (red, green, blue, black), three colour-stimuli congruency conditions, and 7 repetitions for a total of 84 trials randomly sampled. We calculated the median reaction times (RTs) for correct responses in incongruent and congruent trials [50, 51]. The Stroop interference effect was calculated as the difference between the median RTs of the incongruent trials and the congruent trials [incongruent RT – congruent RT] for correct trials only. A larger interference score is indicative of poorer inhibition. We also calculated the proportion of correct responses in incongruent trials because this outcome has been previously linked to the frequency of fatty food consumption [52].

#### Working memory

Working memory is the ability to monitor the relevance of incoming stimuli and update information in memory as required and is implied in goal-shielding (e.g., stick to healthy eating goals). We implemented a backwards digit-span task on Inquisit software [53] because this task has been previously used to investigate executive functioning performance in individuals with excess energy intake [47]. This task required the participants to repeat series of digits of increasing length in reversed order. If participants made a correct response the subsequent trial moved up a level (addition of a digit), if the participants made an incorrect response the subsequent trial moved down a level (removal of a digit). The first trial was a sequence of two visual digits and the task consisted of 14 trials. We calculated the two-error maximum length as the last digit-span a participant got correct before making two consecutive errors and the maximum length i.e., the maximal backward digit span that a participant recalled correctly during all 14 trials.

#### Self-control

The Brief Self-Control Scale (BSCS) was included as a distinct, but conceptually related measure of executive function, as it requires participants to rate their ability to exert control over their behaviour (α = 0.85) [54].

#### Fast-food environment questionnaire

Participants were asked what they believed the aims of the study were (open-text). Participants who mentioned the influence of energy labelling or increased availability of lower energy options on food choices were coded as aim guessers by two independent researchers and discrepancies were resolved by a third researcher. Participants next completed seven questionnaire items to assess the fidelity of the virtual environment, whether the order they made constituted a sufficient amount of food, and whether they were influenced by the energy content of food when choosing. See Additional file 1: Section 4 for a detailed description.

### Procedure

Participants were informed that the study examined food choices at fast-food restaurants. They first answered series of demographic questions including measures of SEP. Participants then completed the virtual fast-food restaurant ordering task. Participants were asked to imagine they were visiting a fast food restaurant for a main meal and to choose their meal from the menu boards, including a main item, a side and a drink. Complete instruction are available in Additional file 1: Section 3. While they were selecting the food items they would like to order, participants were able to navigate between the mains, sides and drinks menu boards and could change selected options before finalising their order. After completing the ordering task, participants next they completed the measures of food choices motivations, self-control, executive functioning and the fast-food environment questionnaire. Participants were then debriefed and paid.

### Statistical analyses

We followed pre-registered analysis protocols (https://osf.io/ajcr6/). The primary analysis for Study 1 and Study 2 was an ANCOVA testing the effect of energy labelling (absent vs. present), availability (baseline vs. increased availability of lower energy options), level of education (continuous variable) and labelling*level of education and availability*level of education interactions on total energy ordered. Analyses were also performed on pooled data from Study 1 and Study 2 to examine the effect of the interventions and of level of education (continuous variable – ANCOVA) or highest educational level (binary, between subjects – ANOVA) on total energy ordered. We also tested whether any participant individual differences (executive functioning, self-control, health motives, fast-food consumption frequency) moderated the effects of availability or energy labelling on total energy ordered.^1^ Data from participants who did not complete the study were not included in primary analyses.^2^ For Study 1 and Study 2, sensitivity analyses were performed by replicating the main analyses 1/ after excluding aim guessers, 2/ substituting the composite variable ‘level of education’ for ‘years in higher education’ as a continuous variable, and 3/ substituting ‘level of education’ by ‘highest educational level’ as a binary variable split into lower vs. higher education level. As secondary analyses for Study 1 and Study 2, we tested whether the effects of the interventions were moderated by alternative measures of SEP (equivalised income, SSS), or fast-food consumption frequency.

All statistical analyses described above were performed using SAS version 9.3 (SAS Institute, Inc., 2012 SAS® 9.3. Cary, NC). Statistical tests level of significance was set at *p* <  0.05 for main and sensitivity analyses, and *p* <  0.01 in secondary analyses to account for multiple testing. To further examine overall evidence, Bayesian analyses were also performed on pooled data as an alternative statistical approach (JASP Version 0.9.2). As opposed to frequentist analyses that can only test whether a null hypothesis can be rejected or not, Bayes factors can determine whether a null hypothesis is supported by the data [55].

### Sample size

A recent meta-analysis showed a statistically significant 7% reduction in energy purchased at restaurants when menus were energy labelled [19]. As previous studies on the effect of an increased availability of lower-energy options on food selection have produced similar or larger effects than kcal labelling [21, 56, 57], we powered the study to be able to detect 7% reductions in energy purchased by intervention conditions. Based on the average energy content of menu item combinations we conservatively estimated a 30% SD (of total energy ordered). We required a sample size of 788 participants for each study (197 per condition) to detect a significant main effect of energy labelling and/or availability or significant interaction effects with SEP, 80% power at α = 0.05 (GPower 3.1).

## Results

### Participants

**Fig. 2:**
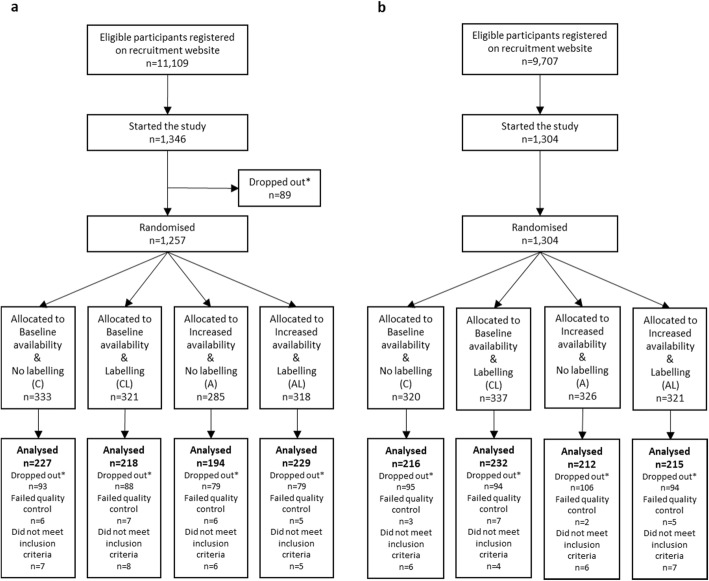
Flow charts. **a** Study 1. **b** Study 2. Legend: *Dropouts were primarily due to problems with software compatibility

Across both studies, 2650 participants started the study and data from 1743 who completed the studies were analysed (Fig. 2). Participants’ characteristics are presented Table 1.

**Table 1:** Participants’ characteristics

	Study 1^a^ (*n* = 868)	Study 2^b^ (*n* = 875)
Age, years, mean (SD)	35.5 (13.4)	36.1 (12.0)
Gender, female, n (%)	419 (48.27)	463 (52.91)
Ethnicity, n (%)		
*White*	789 (90.90)	801 (91.54)
BMI, kg/m2, mean (SD)	26.5 (5.78)	27.1 (5.98)
*Missing, implausible*^*c*^*, n (%)*	16 (1.84)	16 (1.83)
Highest educational level, n (%)		
*No qualification*	17 (1.96)	15 (1.71)
*1–3 GCSEs*	62 (7.14)	52 (5.94)
*4+ GCSEs*	144 (16.59)	119 (13.60)
*A level*	243 (28.00)	286 (32.69)
*Higher education or Bachelor’s degree*	311 (35.83)	330 (37.72)
*Post-Graduate degree*	91 (10.48)	73 (8.34)
Years of higher education, mean (SD)	3.17 (2.63)	3.16 (2.52)
Equivalised income, £, mean (SD)	19,652 (26561)	20,296 (15139)
Subjective socioeconomic status, mean (SD)	4.99 (1.62)	4.95 (1.53)
Student, yes, n (%)	217 (25.00)	32 (3.66)
Fast-food consumption frequency, n (%)		
*Less than once per month*	259 (29.84)	247 (28.23)
*1–3 times per month*	436 (50.23)	456 (52.46)
*1 time per week or more*	173 (19.93)	169 (19.31)
Dieting status, yes, n (%)	119 (13.71)	121 (13.83)

^a^See Additional file 1: Section 5 – Table S4 for study 1 detailed participants’ characteristics. ^b^See Additional file 1: Section 5 – Table S5 for study 2 detailed participants’ characteristics. ^c^BMI implausible values: BMI > 10 or BMI < 60 [58]

### Effect of the interventions and level of education on total energy ordered

On average, the participants selected 881 ± 217 kcal (mean ± SD) in Study 1 and 909 ± 236 kcal (mean ± SD) in Study 2. Table 2 reports total energy ordered in each condition.

**Table 2:** Total energy ordered by experimental condition

	Study 1			Study 2		
	n	Mean	SD	n	Mean	SD
Baseline availability & No labelling (C)	227	927	177	216	961	210
Baseline availability & Labelling (CL)	218	911	192	232	921	206
Increased availability & No labelling (A)	194	843	239	212	879	258
Increased availability & Labelling (AL)	229	839	243	215	874	257

Study 1 showed a significant effect of availability but no significant effect of labelling and level of education. There were also no significant interactions (Table 3). Study 2 produced the same results (Table 3). Results were unchanged in sensitivity analyses and secondary analyses. See Additional file 1: Section 6 – Table S6.

**Table 3:** ANCOVA models, dependant variable: total energy ordered

Model	*F*	*p*	partial η^2^
Study 1 (*n* = 868)			
availability	28.55	< 0.001	0.0321
labelling	0.47	0.494	0.0005
level of education	0.69	0.406	0.0008
availability*level of education	2.34	0.127	0.0027
labelling*level of education	0.52	0.471	0.0006
Study 2 (*n* = 875)			
availability	16.29	< 0.001	0.0184
labelling	2.01	0.157	0.0023
level of education	2.92	0.088	0.0033
availability*level of education	0.02	0.875	< 0.0001
labelling*level of education	0.17	0.680	0.0002
Pooled data (*n* = 1743)			
availability	43.35	< 0.001	0.0244
labelling	2.47	0.116	0.0014
level of education	0.31	0.575	0.0002
availability*level of education	1.27	0.260	0.0007
labelling*level of education	0.05	0.816	< 0.0001
study	6.95	0.009	0.0040

**Fig. 3:**
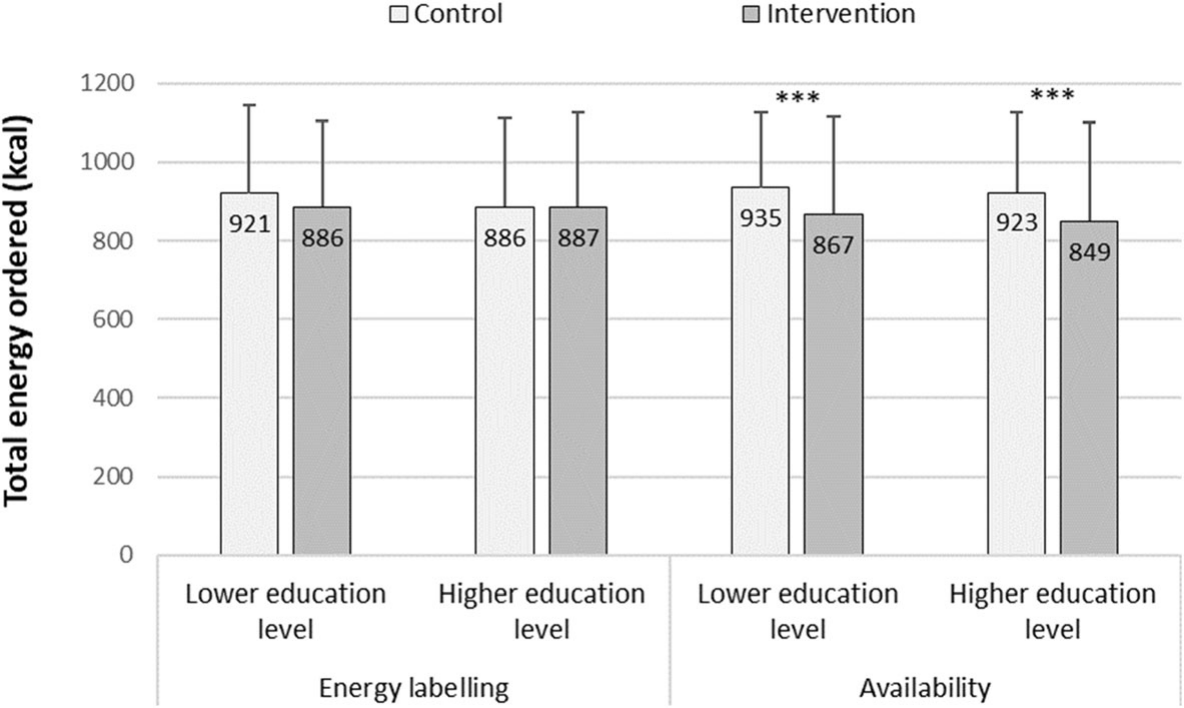
Mean (+ SD) of total energy ordered for pooled data. Legend: Energy labelling control: C and A conditions, energy labelling intervention: CL and AL conditions, availability control: C and CL conditions, availability intervention: A and AL conditions. Lower education level: A level or below, higher education level: above A-level. *** *p* < 0.001 least square means post-hoc tests

To evaluate the overall body of evidence from both studies, analytic data from studies 1 and 2 were combined (*n* = 1743) and statistical analyses were run on pooled data adjusting for the origin of each participant’s data (study: 1 vs. 2 included as an independent variable). The combined dataset increased the power of our main analysis from 0.80 to 0.99 (GPower 3.1). In line with the results of the individual studies, there was a significant effect of availability, whereby increasing the availability of lower energy options resulted in 71 fewer kcal ordered (*p* < 0.001, partial η^2^ = 0.0244). Eighteen fewer kcals were ordered in the presence vs. absence of energy labelling, but this not was significant (*p* = 0.116, partial η^2^ = 0.0014). Education level did not significantly interact with either intervention (Table 3 & Fig. 3). Results were unchanged when substituting level of education (continuous) with highest educational level (binary). See Additional file 1: Section 7 – Table S8. Bayes factors were computed to compare the likelihood of total energy pooled data under several models (see Additional file 1: Section 7 – Table S9). Bayes factors analysis confirmed the results obtained with frequentist statistical methods: only a model including availability as a predictor of total energy ordered was more likely than a null model (BF_10_ = 8.329e+ 7).

We also tested whether any participant individual differences (executive functioning, self-control, health motives, fast-food consumption frequency) moderated the effects of availability or energy labelling using pooled data and found no evidence in support of this. See Additional file 1: Section 7 – Table S10. For completeness and to aid further research, the relationships between SEP, executive function (and the reliability of these measures), health motives and energy ordered using pooled data are reported in Additional file 1: Section 7 – Table S11 & Table S12.

### Fast-food environment questionnaire

In Study 1 and Study 2, participants questionnaire responses did not differ based on experimental condition, indicated that overall the virtual fast-food environment was representative of a real-world fast-food restaurant and participants were satisfied with their meal choices. In Study 1, a third of participants (31%) disagreed that the range of food and drink products in the virtual fast-food restaurant items was acceptable. After increasing the range of food times in Study 2, the majority of participants (83%) believed the range of food and drink options was acceptable. See Additional file 1: Section 4 – Table S3. In addition, participants tended to report that their choices were not influenced by how many calories they thought were in menu options and this did not differ between experimental conditions.

## Discussion

Across two studies we examined the effect of increasing the availability of lower energy menu options and providing menu energy labelling on total energy ordered by participants of higher and lower SEP in a virtual fast-food restaurant. Increasing the availability of lower energy options (from 25 to 75%) resulted in participants ordering meals with statistically significantly less energy on average (− 71 kcal, 7.6% reduction). Menu energy labelling had no statistically significant effect on total energy ordered (− 18 kcal, 2.0% reduction). We found no evidence that participant SEP moderated the effect of availability or energy labelling on total energy ordered, irrespective of whether SEP was based on education level, household income or subjective (participant perceived) SEP.

The effect of increasing availability of lower energy options on food choice is consistent with other studies [57, 59] and the lack of moderation by SEP is consistent with the proposition that ‘structural-based’ interventions to improve nutrition are likely to be equitable [32]. Based on mixed findings to date, we tentatively predicted that there would be a small effect of energy labelling on total energy ordered and that this effect would be predominantly observed among participants from higher SEP. However, there was no significant effect of menu energy labelling in either study or in a larger pooled analysis. In the pooled analysis a small non-significant reduction in energy ordered was observed, which is consistent with the conclusions of recent systematic reviews; energy labelling has no measurable impact [60, 61] or only a very small effect on amount of energy ordered when eating out [19]. Furthermore, we found no evidence that energy labelling was any more effective when there was an increased range of lower energy menu options (interaction between interventions). Given that there is need to consider whether policies can ameliorate SEP inequalities in obesity [62], the present findings suggest that neither increasing availability of lower energy options nor energy labelling in fast food restaurants are likely to directly achieve this aim by altering the food choices of lower vs. higher SEP populations.

We found some evidence that participants with a higher SEP tended to report being more strongly motivated by weight control, health and calories when making food choices and had greater executive functioning performance than lower SEP participants (reported in Additional file 1). However, we failed to find evidence in support of the proposition that energy labelling is more effective in changing the diet of higher as opposed to lower SEP participants [22]. We also examined other potential moderators of the impact of energy labelling or availability on total energy ordered (executive functioning, self-control, healthiness and weight control motivation, fast-food consumption frequency), but found no supporting evidence (reported in Additional file 1). The lack of effect of energy labelling observed may relate to the restaurant environment used. There is mixed evidence on the impact of energy labelling on purchases made in fast-food restaurants [63, 64]. Fast-food purchases may be perceived as being an indulgence or ‘treat’ and therefore customers do not base their choices on health. In line with this, we found that only a small minority of participants reported being influenced by the energy content of the food in labelling condition in the present studies (23% in Study 1, 20% Study 2). In addition, the food items used in both studies were from a popular fast food chain in the UK. The effect of energy labelling on energy ordered may depend on existing knowledge about the energy content of menu items, whereby energy labelling may only impact behaviour when consumers are confronted with nutrition information that does not align with their expectations [65]. Future research is needed to explore further the effect of these interventions in different settings (e.g., full-service restaurants) and to test whether menu options people are unaware of the energy content of are more likely to be affected by energy labelling.

Strengths of the present studies included pre-registration, high statistical power and balanced recruitment of a large number of participants from higher and lower SEPs. However, the participants were recruited through a self-selected online panel which limits the generalisability of the results. Our main measure of SEP was education level. Although results were consistent irrespective of SEP measure used (education, income, perceived SEP) we did not examine all possible SEP measures (e.g. occupation), but we are not aware of a convincing hypothesis of why the effects of availability or energy labelling would be moderated by other measures of SEP. A strength of using virtual environments is that hypotheses can be tested under tightly controlled experimental conditions. The virtual fast-food restaurant used was created for the present studies and has therefore not been validated. We based the restaurant design and menu information (including meal choices, energy content, price) on a popular UK fast-food restaurant and participants reported that the food items in the virtual environment were common in fast-food restaurants, and that there were food items they would have normally ordered in the real world. We also found evidence that participants’ food choices in the virtual environment were similar to what would be expected in everyday life, e.g., as shown in other research [30, 66], participants tended to order less energy overall if they reported being motivated by health when making everyday food choices. Although other research has shown that food choice tasks using virtual environments are valid [67, 68], participants did not spend their own money and did not have to consume what they ordered. Moreover, the food items were from one fast food chain and generalizing results to other eating out settings should be done with caution. Thus, replicating the present findings in diverse real-world settings and measuring actual choice behaviour in fast-food restaurants is now needed.

## Conclusion

In a virtual fast-food environment, energy labelling was ineffective in reducing total energy ordered for participants of both higher and lower SEP. Increasing the availability of lower energy options had an equitable effect, reducing total energy ordered in participants from higher and lower SEP.

## Supplementary information

**Additional file 1.** All additional materials and data.

## Data Availability

The datasets analysed during the current studies are available on the Open Science Framework project page https://osf.io/ajcr6/.

## Abbreviations

A: ‘increased availability of lower energy options’ and ‘no energy labelling’
AL: ‘increased availability of lower energy options’ and ‘energy labelling’
BMI: Body mass index
C: ‘baseline availability’ and ‘no energy labelling’
CL: ‘baseline availability’ and ‘energy labelling’
HE: Higher energy
LE: Lower energy
SD: Standard deviation
SEP: Socioeconomic position

## Footnotes

1 As detailed in the results section, we did not find any interaction between SEP and labelling. Consequently the moderated mediation analysis that was planned to investigate the potential mediators of the differential effect of labelling in lower vs. higher SEP participants was not performed (see pre-registered analysis plan: https://osf.io/ajcr6/).

2 Results remained the same when including available data from participants who dropped out. See Additional file – **Section 6 – Tables S6 and S7**.
