# Supplementary material for: Socioeconomic position and the impact of increasing availability of lower energy meals vs. menu energy labelling on food choice: two randomized controlled trials in a virtual fast-food restaurant
Source: Int J Behav Nutr Phys Act. 2020 Jan 31;17:10. doi: 10.1186/s12966-020-0922-2 (PMC6995045; doi:10.1186/s12966-020-0922-2)
Supplement: Supplementary file 1 — Additional file 1. All additional materials and data. [file 12966_2020_922_MOESM1_ESM.docx]

**Additional file 1**

**Table of content**

[**1.** **Quality control (attention check) questions** 3](#_Toc29292336)

[**2.** **Highest educational qualification measure** 3](#_Toc29292338)

**3**[**. Description of the virtual fast food ordering task** 3](#_Toc29292339)

**4**[**. Fast-food environment questionnaire** 7](#_Toc29292340)

[*4.1.* *Study 1 fast-food environment questionnaire analysis* 8](#_Toc29292341)

[*4.2.* *Study 2 fast food environment questionnaire analysis* 9](#_Toc29292342)

[**5.** **Detailed participants’ characteristics overall and by intervention condition** 10](#_Toc29292343)

[**6.** **Sensitivity, secondary and additional analyses** 12](#_Toc29292344)

[**7.** **Pooled analyses** 15](#_Toc29292345)

[*7.1.* *Effect of the interventions and level of education on total energy ordered* 15](#_Toc29292346)

[*7.2.* *Bayesian pooled analyses* 16](#_Toc29292347)

[*7.3.* *Moderation of interventions by participant individual differences* 17](#_Toc29292348)

[*7.4.* *Relationships between SEP, executive function, food choice motives and energy ordered* 19](#_Toc29292349)

[**8.** **References** 20](#_Toc29292350)

**Tables of figures**

[**Figure S1.** Images of the menu boards for the main in Study 1. 7](#_Toc27581947)

[**Figure S2.** Images of the menu boards for the main in Study 2. 7](#_Toc27581948)

**Table of tables**

[**Table S1.** Food items included on Study 1 menu boards in descending order (kcal). Items in bold are the same across all experimental conditions. 7](#_Toc22574068)

[**Table S2.** Food items included on Study 2 menu boards in descending order (kcal). Items in bold are the same across all experimental conditions. 8](#_Toc22574069)

[**Table S3**. Descriptive analyses for each item of the debriefing questionnaire 10](#_Toc22574070)

[**Table S4.** Study 1 detailed participants’ characteristics overall and by intervention condition 12](#_Toc22574071)

[**Table S5.** Study 2 detailed participants’ characteristics overall and by intervention condition 13](#_Toc22574072)

[**Table S6.** ANCOVA models for sensitivity, secondary and additional analyses for Study 1, dependent variable: total energy ordered (n=868 unless otherwise specified) 15](#_Toc22574073)

[**Table S7.** ANCOVA models for sensitivity, secondary and additional analyses for Study 2, dependent variable: total energy ordered (n=875 unless otherwise specified) 16](#_Toc22574074)

[**Table S8.** Statistical models for pooled analyses, dependent variable: total energy ordered (n=1,743) 18](#_Toc22574075)

[**Table S9.** ANCOVA models testing the effect of the interventions and potential moderators (continuous variables) on total energy ordered (n=1,743) 19](#_Toc22574076)

[**Table S10.** Bayes factors for comparison of the likelihood of total energy data under several models 19](#_Toc22574077)

[**Table S11.** Split-half estimates for the main measures of the Stroop and digit-span tasks (n=1,743) 21](#_Toc22574078)

[**Table S12.** Partial correlation coefficients between level of education, total energy ordered and executive function, self-control, food choice motives adjusted for study (n=1,743) 22](#_Toc22574079)

1. **Quality control (attention check) questions**

We included two quality control (attention check) questions in Study 1 and Study 2. The first quality control item was “This is an attention check. How many times have you visited the planet Mars?” and possible responses were ‘Several times’, ‘Just once’ and ‘Never’; and the second item was “This is an attention check. Please choose the answer 2 ‘Not important’” with possible responses on a 7-point scale from 1 ‘Not at all important’ to 7 ‘Very important’. Participants were excluded if they gave any responses other than ‘Never’ to the first item and other than ‘Not important’ to the second item.

1. **Highest educational qualification measure**

Highest educational qualification was measured using the question “What is your highest educational qualification? If you are a student please select the diploma being studied for.” Participants selected one of the following options, coded from 1 to 9: No formal qualifications, 1-3 GCSEs or equivalent, 4+ GCSEs or equivalent, A level or equivalent, Certificate of higher education (CertHE) or equivalent, Diploma of higher education (DipHE) or equivalent, Bachelor’s degree or equivalent, Master’s degree or equivalent, Doctoral degree or equivalent.

1. **Description of the virtual fast food ordering task**

Before completing the virtual fast food ordering task, participants read the following instructions: “We would like you to imagine that you are visiting a fast food restaurant for a main meal (this can be lunch or dinner, whichever you would be most likely to visit a fast food restaurant for). On the next page, you will be at the door of the fast food restaurant. You will be able to click on the door to enter the store. You can look around the restaurant by holding the mouse clicker down and move the mouse around. Use the red circles on the ground to navigate in the fast food environment. Click on the counter to move there and then click on the order screen to be able to view the menu options. You will be asked to choose your meal from the menu boards, including a main item, a side and a drink. When making your meal choice, try to imagine you actually are in a fast food restaurant and choose food items that you would eat. Before starting, make sure to be in a quiet area and put your speakers on.”


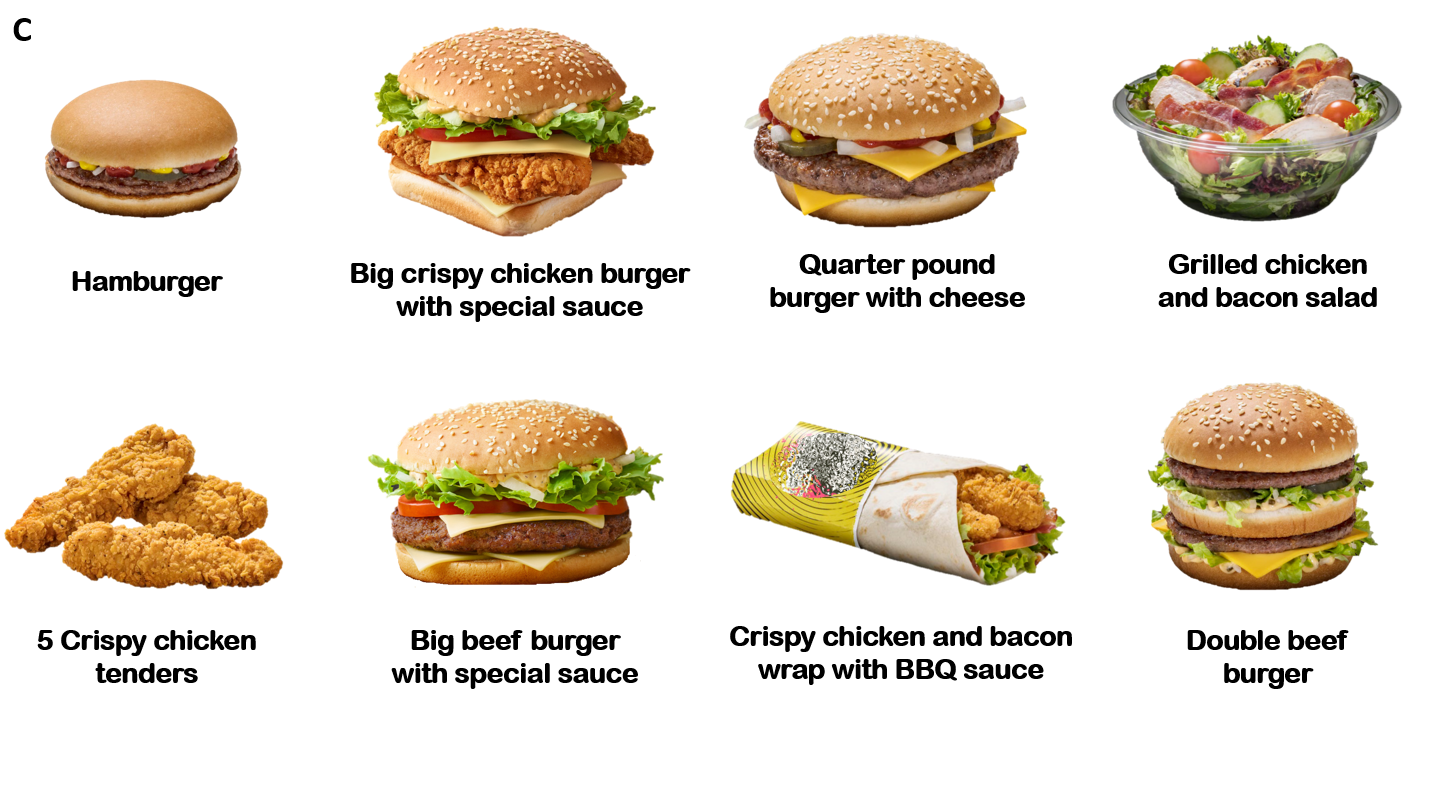

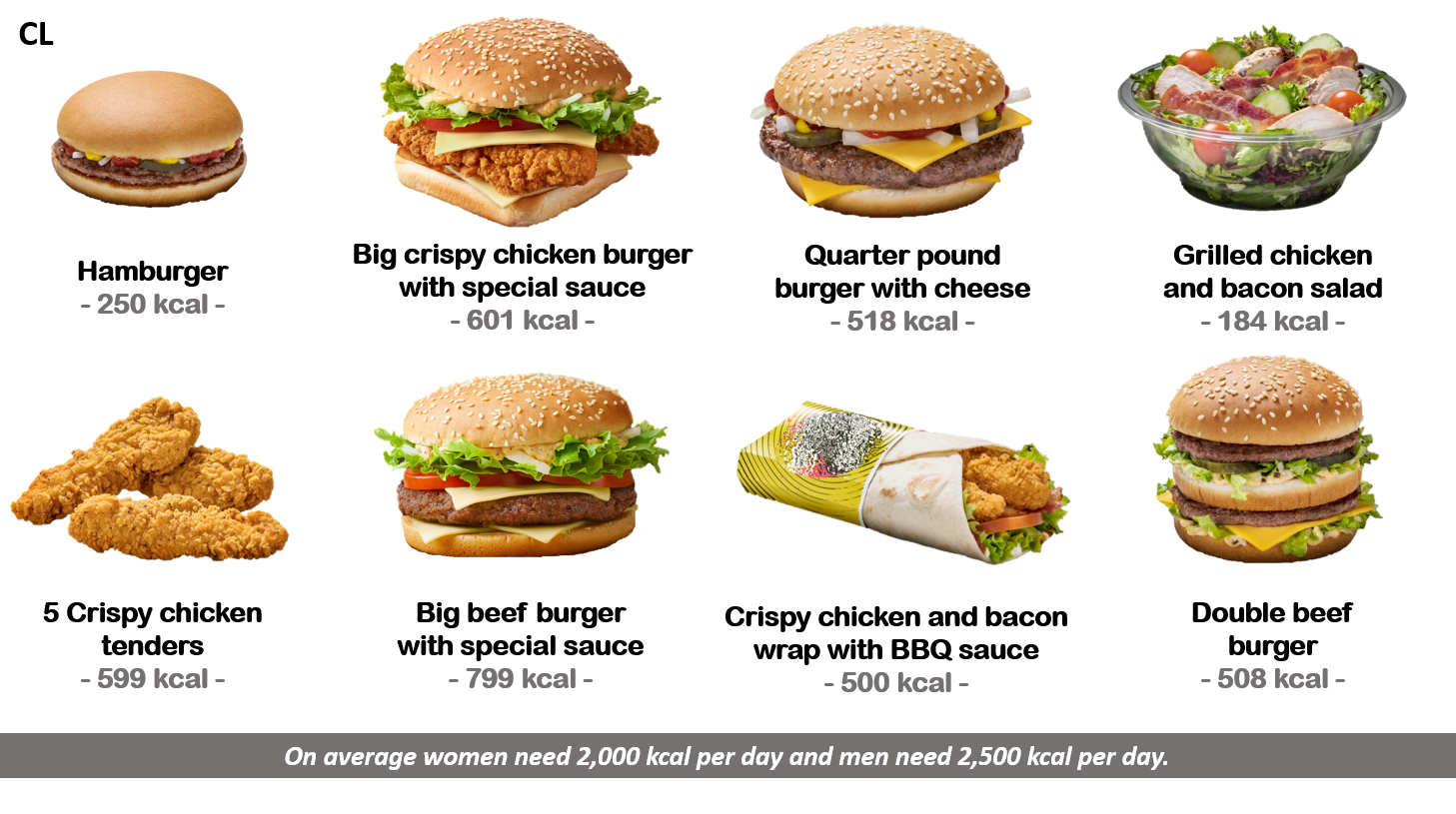

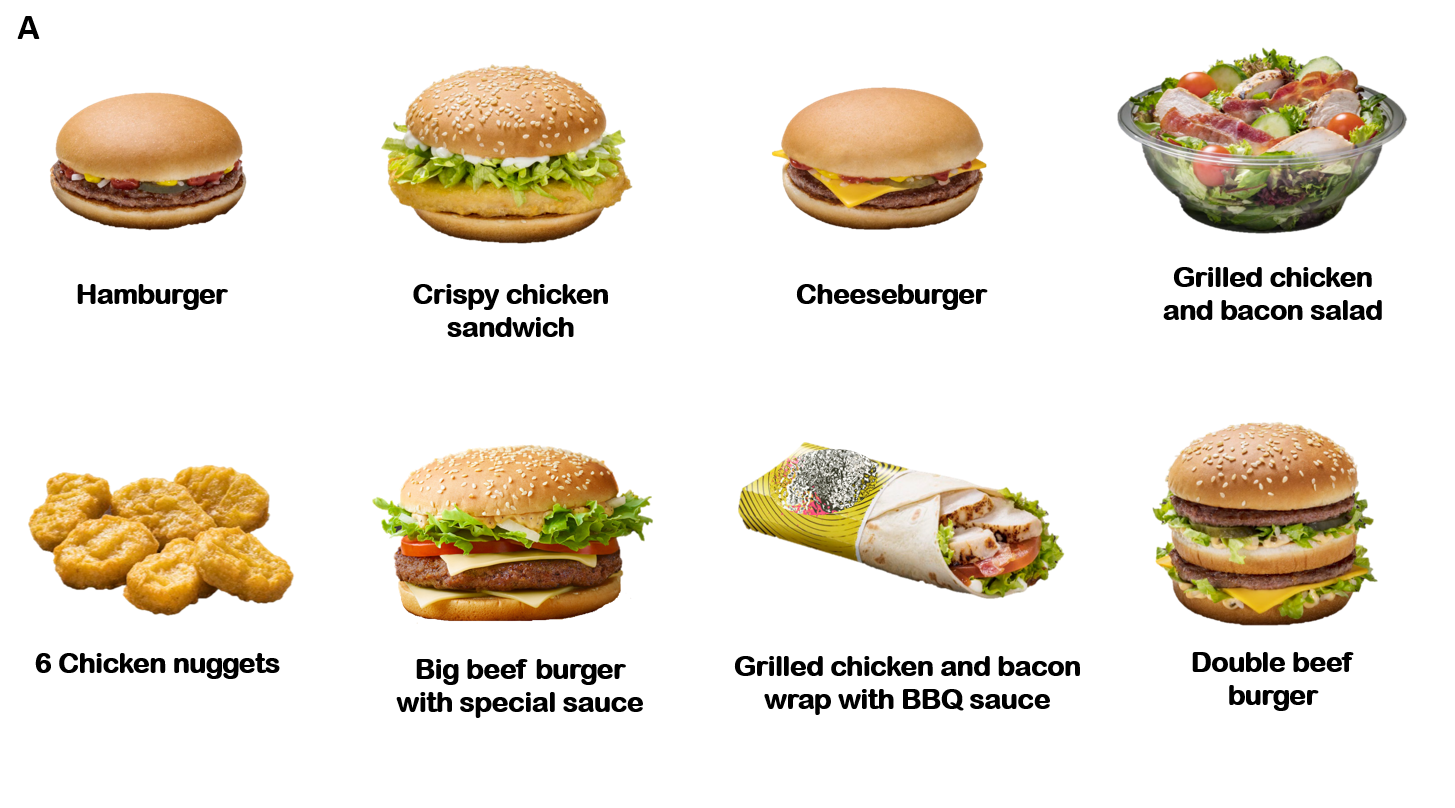

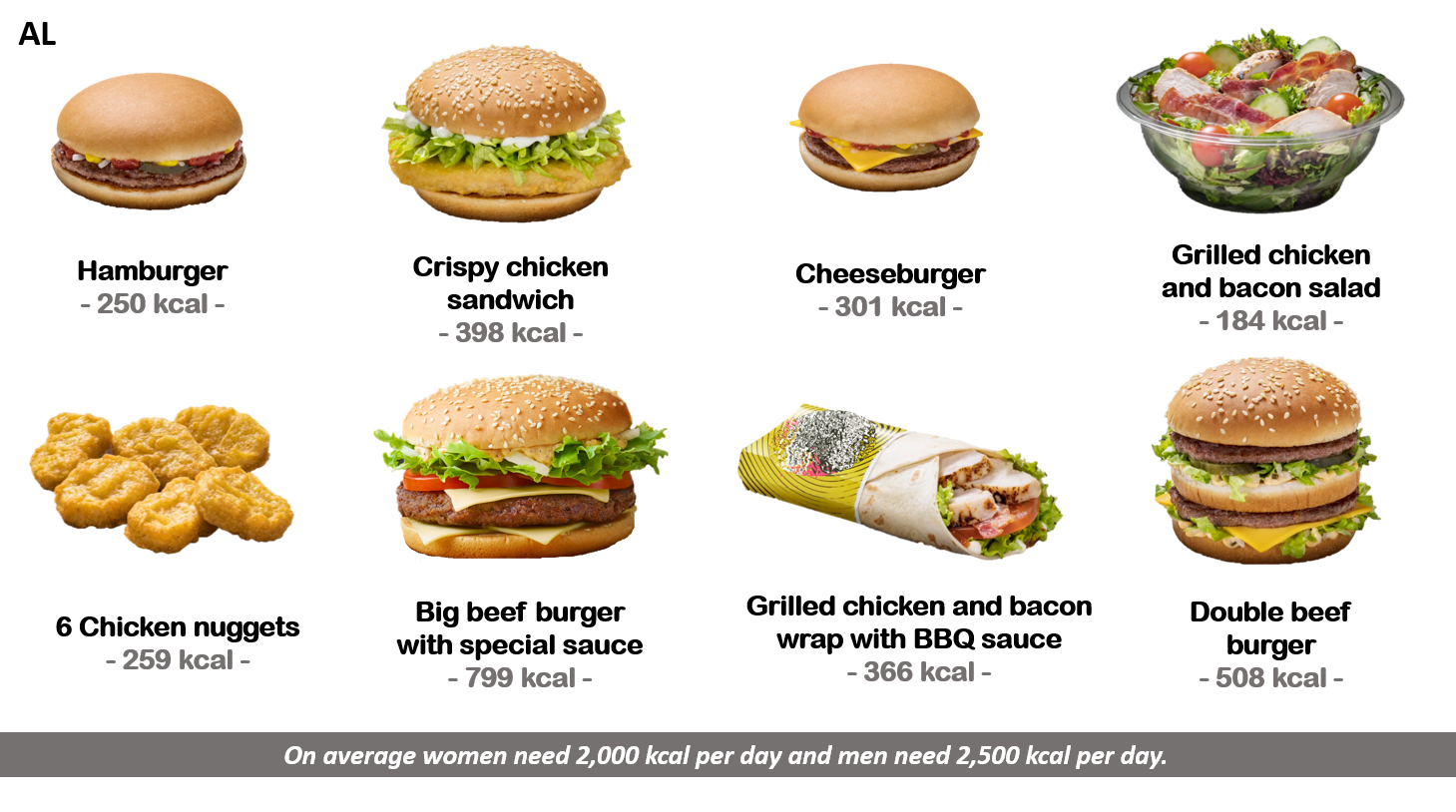


**Figure S1.** Images of the menu boards for the mains in Study 1.

*Legend:* C: ‘baseline availability’ and ‘no energy labelling’, CL: ‘baseline availability’ and ‘energy labelling’, A: ‘increased availability of lower energy options’ and ‘no energy labelling’, AL: ‘increased availability of lower energy options’ and ‘energy labelling’.


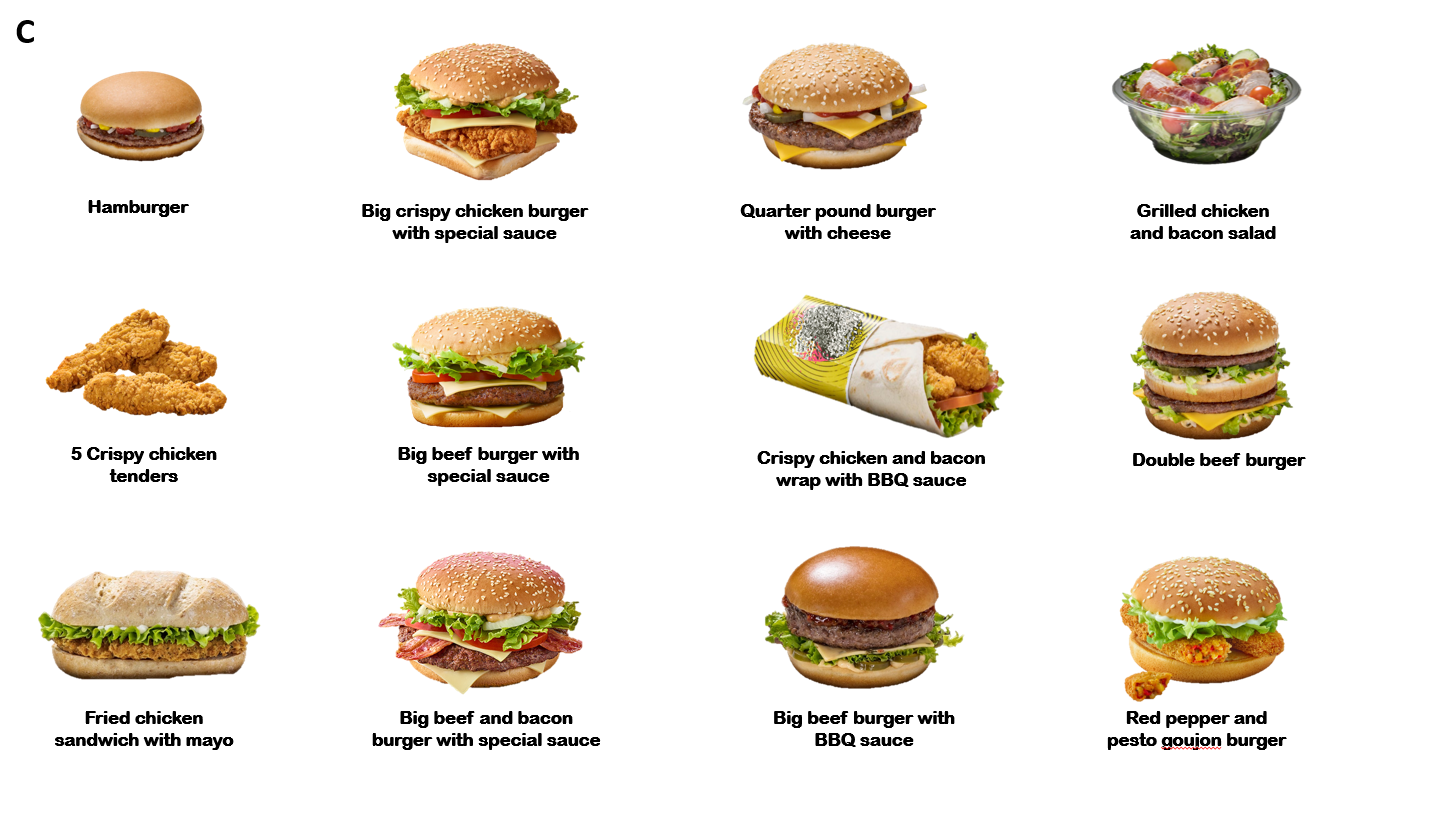

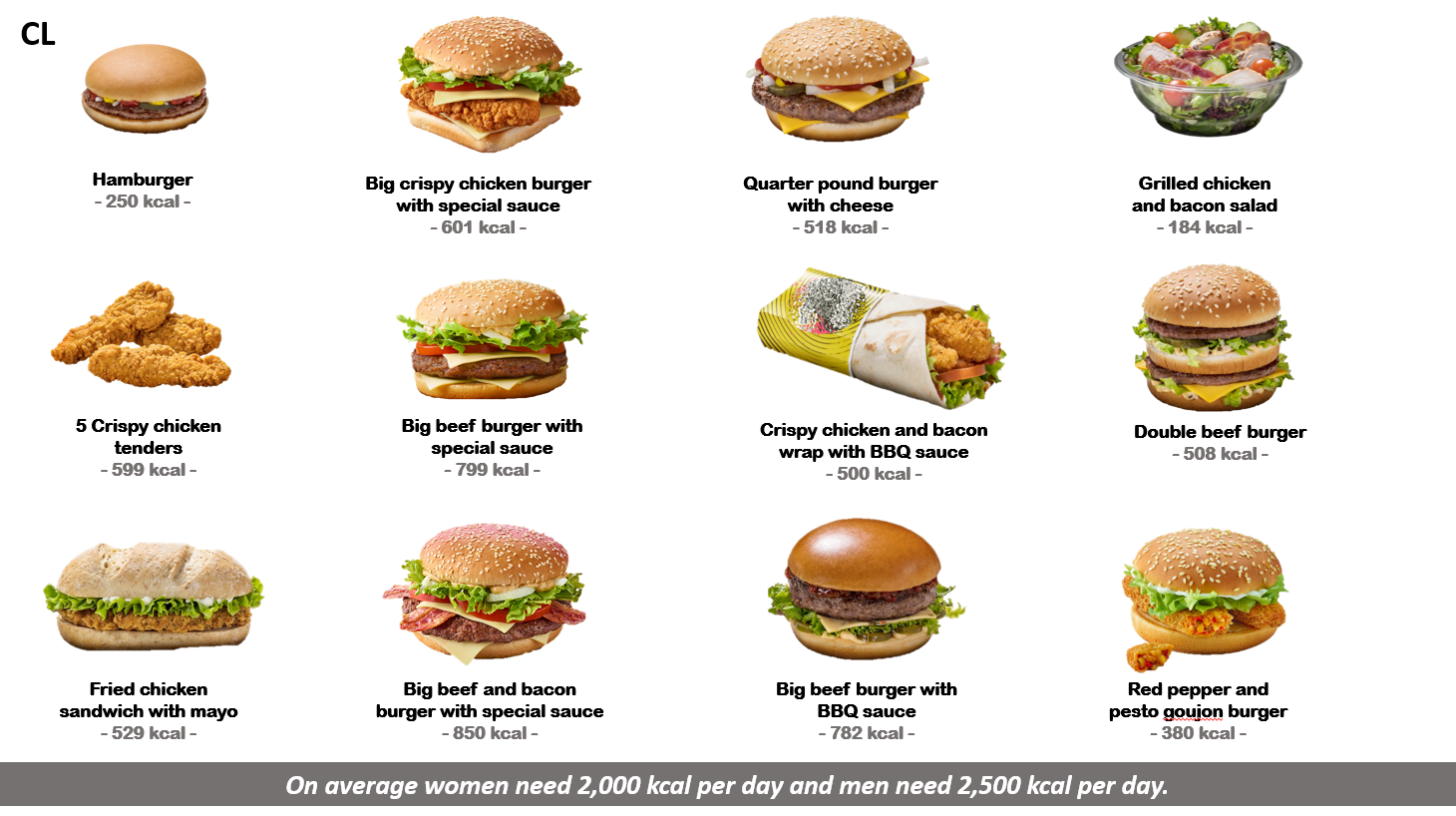

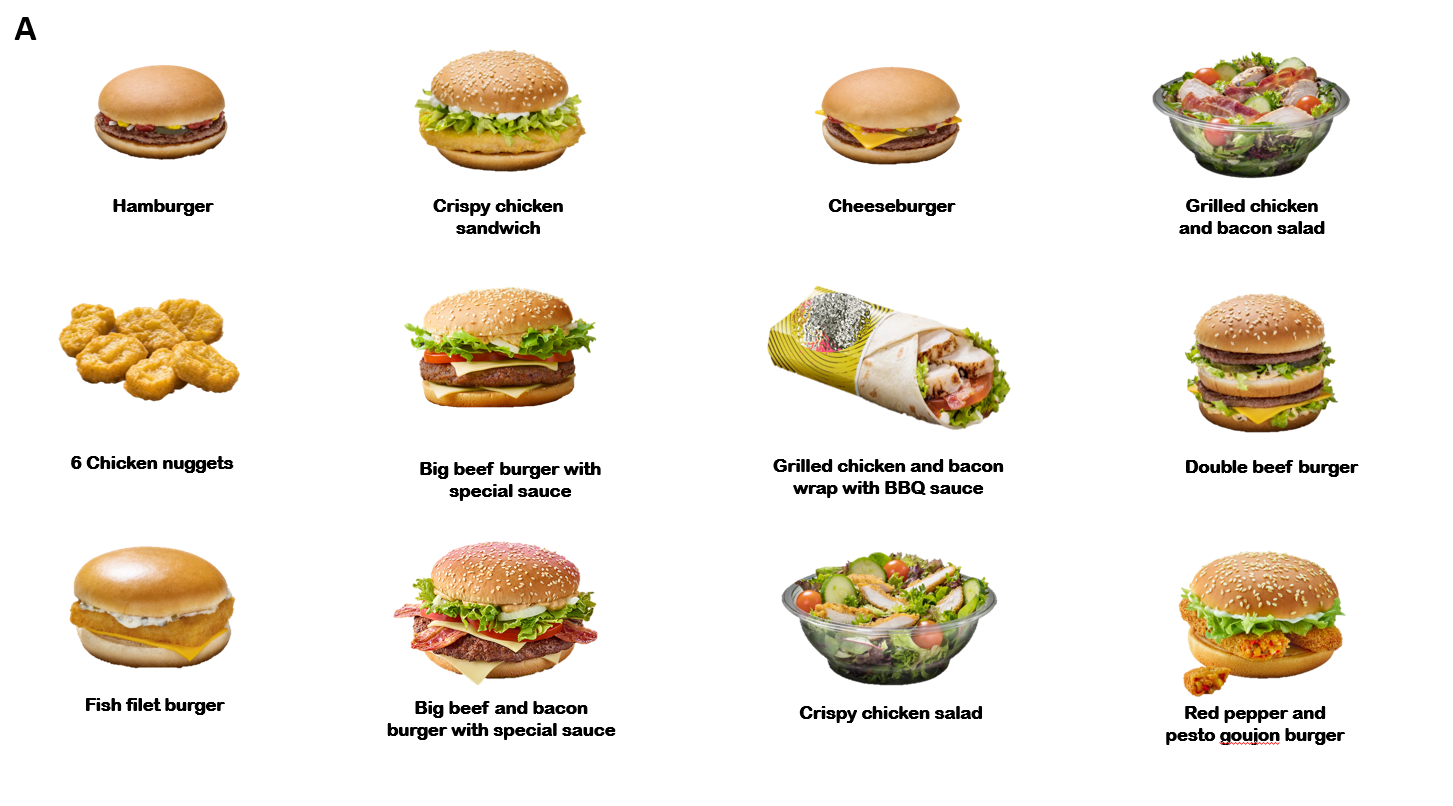

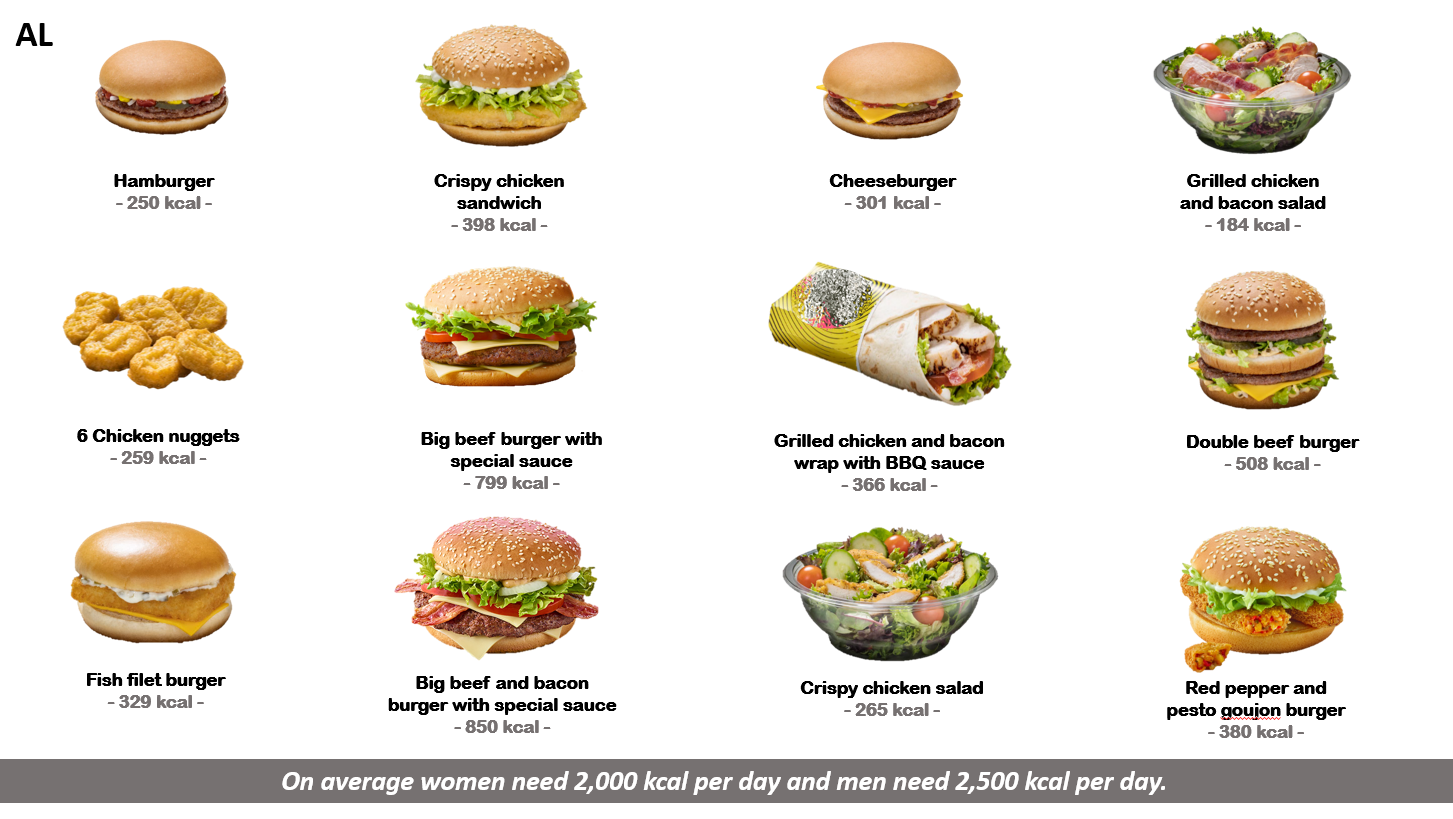


**Figure S2.** Images of the menu boards for the mains in Study 2.

*Legend:* C: ‘baseline availability’ and ‘no energy labelling’, CL: ‘baseline availability’ and ‘energy labelling’, A: ‘increased availability of lower energy options’ and ‘no energy labelling’, AL: ‘increased availability of lower energy options’ and ‘energy labelling’.

**Table S1.** Food items included on Study 1 menu boards in descending order (kcal). Items in bold are the same across all experimental conditions.

|  | **C & CL** | | | **A & AL** | | |
| --- | --- | --- | --- | --- | --- | --- |
|  | **Names** | **Kcal** | **HE/**  **LE** | **Names** | **Kcal** | **HE/**  **LE** |
| **Mains** | **Big beef burger special sauce**  Big crispy chicken burger special sauce  5 Crispy chicken tenders  Quarter pound burger with cheese  **Double beef burger**  Crispy chicken and bacon wrap BBQ  **Hamburger**  **Grilled Chicken and Bacon Salad** | **799**  601  599  518  **508**  500  **250**  **184** | **HE**  HE  HE  HE  **HE**  HE  **LE**  **LE** | **Big beef burger special sauce**  **Double beef burger**  Crispy chicken sandwich  Grilled chicken and bacon wrap BBQ  Cheeseburger  6 Chicken nuggets  **Hamburger**  **Grilled Chicken and Bacon Salad** | **799**  **508**  388  366  301  259  **250**  **184** | **HE**  **HE**  LE  LE  LE  LE  **LE**  **LE** |
| **Sides** | **Medium fries**  Cheese dippers  Small fries  **Side salad** | **337**  257  237  **18** | **HE**  HE  HE  **LE** | **Medium fries**  Pineapple stick  Baby carrots  **Side salad** | **337**  37  34  **18** | **HE**  LE  LE  **LE** |
| **Drinks** | **Cola**  ***Large***  ***Medium***  ***Small***  Orange soda  *Large*  *Medium*  *Small*  Mixed fruit squash  *Large*  *Medium*  *Small*  **Diet cola**  ***Large***  ***Medium***  ***Small*** | **212**  **170**  **106**  95  76  48  83  67  42  **2**  **1**  **1** | **HE**  **HE**  **HE**  HE  HE  HE  HE  HE  HE  **LE**  **LE**  **LE** | **Cola**  ***Large***  ***Medium***  ***Small***  Diet lemonade  *Large*  *Medium*  *Small*  Cola zero  *Large*  *Medium*  *Small*  **Diet cola**  ***Large***  ***Medium***  ***Small*** | **212**  **170**  **106**  5  4  3  2  1  1  **2**  **1**  **1** | **HE**  **HE**  **HE**  LE  LE  LE  LE  LE  LE  **LE**  **LE**  **LE** |

C: ‘baseline availability’ and ‘no energy labelling’, CL: ‘baseline availability’ and ‘energy labelling’, A: ‘increased availability of lower energy options’ and ‘no energy labelling’, AL: ‘increased availability of lower energy options’ and ‘energy labelling’, LE: ‘lower energy’, HE: ‘higher energy’.

**Table S2.** Food items included on Study 2 menu boards in descending order (kcal). Items in bold are the same across all experimental conditions.

|  | **C & CL** | | | **A & AL** | | |
| --- | --- | --- | --- | --- | --- | --- |
|  | **Names** | **Kcal** | **HE/**  **LE** | **Names** | **Kcal** | **HE/**  **LE** |
| **Mains** | **Big beef and bacon burger special sauce**  **Big beef burger special sauce**  Big beef burger BBQ  Big crispy chicken burger special sauce  5 Crispy chicken tenders  Fried chicken sandwich with mayo  Quarter pound burger with cheese  **Double beef burger**  Crispy chicken and bacon wrap BBQ  **Red pepper & pesto goujon burger**  **Hamburger**  **Grilled Chicken and Bacon Salad** | **850**  **799**  782  601  599  529  518  **508**  500  **380**  **250**  **184** | **HE**  **HE**  HE  HE  HE  HE  HE  **HE**  HE  **LE**  **LE**  **LE** | **Big beef and bacon burger special sauce**  **Big beef burger special sauce**  **Double beef burger**  Crispy chicken sandwich  **Red pepper & pesto goujon burger**  Grilled chicken and bacon wrap BBQ  Fish filet burger  Cheeseburger  Crispy chicken salad  6 Chicken nuggets  **Hamburger**  **Grilled Chicken and Bacon Salad** | **850**  **799**  **508**  388  **380**  366  329  301  265  259  **250**  **184** | **HE**  **HE**  **HE**  LE  **LE**  LE  LE  LE  LE  LE  **LE**  **LE** |
| **Sides** | **Medium fries**  **Cheese dippers**  Small fries  Cheese wedges  Mozarella sticks  **Side salad**  **Cucumber sticks** | **337**  **257**  237  233  190  **18**  **12** | **HE**  **HE**  HE  HE  HE  **LE**  **LE** | **Medium fries**  **Cheese dippers**  Apple and grapes  Pineapple stick  Baby carrots  **Side salad**  **Cucumber sticks** | **337**  **257**  46  37  34  **18**  **12** | **HE**  **HE**  LE  LE  LE  **LE**  **LE** |
| **Drinks** | **Mango and pineapple smoothie**  ***Large***  ***Regular***  **Cola**  ***Large***  ***Medium***  ***Small***  Orange juice  Orange soda  *Large*  *Medium*  *Small*  Mixed fruit squash  *Large*  *Medium*  *Small*  **Diet cola**  ***Large***  ***Medium***  ***Small***  **Still mineral water** | **236**  **187**  **212**  **170**  **106**  108  95  76  48  83  67  42  **2**  **1**  **1**  **0** | **HE**  **HE**  **HE**  **HE**  **HE**  HE  HE  HE  HE  HE  HE  LE  **LE**  **LE**  **LE**  **LE** | **Mango and pineapple smoothie**  ***Large***  ***Regular***  **Cola**  ***Large***  ***Medium***  ***Small***  Apple and blackcurrant drink  Diet lemonade  *Large*  *Medium*  *Small*  Cola zero  *Large*  *Medium*  *Small*  **Diet cola**  ***Large***  ***Medium***  ***Small***  **Still mineral water** | **236**  **187**  **212**  **170**  **106**  10  5  4  3  2  1  1  **2**  **1**  **1**  **0** | **HE**  **HE**  **HE**  **HE**  **HE**  LE  LE  LE  LE  LE  LE  LE  **LE**  **LE**  **LE**  **LE** |

C: ‘baseline availability’ and ‘no energy labelling’, CL: ‘baseline availability’ and ‘energy labelling’, A: ‘increased availability of lower energy options’ and ‘no energy labelling’, AL: ‘increased availability of lower energy options’ and ‘energy labelling’

1. **Fast-food environment questionnaire**

At the end of Study 1 and Study 2 participants completed seven questionnaire items to assess how valid/realistic they believed the virtual environment to be (validity dimension, five items), whether the order they made constituted a sufficient amount of food (compensation dimension, 1 item) and whether they were influenced by the energy content of food when choosing (kcal influence dimension, 1 item) (**Table S3**). Answers to these seven items were coded as: 1 = Strongly disagree; 2 = Disagree; 3 = Slightly disagree; 4 = Neutral; 5 = Slightly agree; 6 = Agree; 7 = Strongly agree. Three ANCOVAs were performed on participants’ answers to examine whether labelling, availability and level of education influenced the 1/ validity, 2/ compensation, or 3/ kcal influence dimensions.

**Table S3**. Descriptive analyses for each item of the debriefing questionnaire

|  |  | **Study 1**  **(n=868)** | | | **Study 2**  **(n=875)** | | |
| --- | --- | --- | --- | --- | --- | --- | --- |
| **Dimension** | **Items** | **Mean (SD)^a^** | **Disagree^b^ (%)** | **Agree^c^ (%)** | **Mean (SD)^a^** | **Disagree^b^ (%)** | **Agree^c^ (%)** |
| Validity | *1. The food and drink items available in the menu boards are common in fast-food restaurants* | 6.38 (0.78) | 1 | 98 | 6.37 (0.78) | 1 | 98 |
|  | *2. The food and drink items I picked for my meal in the virtual fast-food restaurant would be something that I would normally order in the real world* | 6.06 (1.19) | 5 | 91 | 6.21 (1.04) | 3 | 94 |
|  | *3. I am satisfied with the food items I picked for my meal in the virtual fast-food restaurant.* | 5.91 (1.08) | 4 | 90 | 6.00 (1.03) | 4 | 94 |
|  | *4. I would have felt satisfied after consuming the food items I picked for my meal in the virtual fast-food restaurant.* | 5.60 (1.28) | 8 | 85 | 5.67 (1.26) | 8 | 86 |
|  | *7. There was an acceptable range of food and drink items in the virtual fast-food restaurant.* | 4.61 (1.73) | 31 | 62 | 5.24 (1.54) | 17 | 76 |
| Compensation | *5. To feel sufficiently full I would require more food after consuming the food items I picked for my meal in the virtual fast-food restaurant.* | 3.38 (1.92) | 58 | 34 | 3.38 (1.85) | 56 | 33 |
| Kcal influence | *6. The choices I made in the virtual fast-food restaurant were influenced by how many calories I thought were in the menu options available.* | 2.65 (1.72) | 73 | 20 | 2.55 (1.65) | 75 | 18 |

^a^Range: 1: strongly disagree; 2: disagree; 3: slightly disagree; 4: neutral; 5:slightly agree; 6: agree; 7: strongly agree. ^b^1 to 3. ^c^5 to 7

- 1. *Study 1 fast-food environment questionnaire analysis*

Participants tended to rate the virtual fast-food environment highly on the validity dimension. A validity score was calculated as the mean of the five items in this dimension (McDonald’s ω = 0.732). There was no significant effect of availability, *F*(1, 864) = 0.25, *p* = 0.615, partial η^2^ = 0.0003, and labelling, *F*(1, 864) = 0.66, *p* = 0.418, partial η^2^ = 0.0008, but a significant effect of level of education, *F*(1, 864) = 11.23, *p* < 0.001, partial η^2^ = 0.0128, on the validity dimension. Participants with a higher level of education tended to rate the virtual fast-food environment lower on the validity dimension than participants with a lower level of education.

Participants tended to disagree that they would have required more food after consuming their selected meal (compensation dimension). There was no significant effect of availability, *F*(1, 864) = 2.11, *p* = 0.147, partial η^2^ = 0.0024, and labelling, *F*(1, 864) = 3.19, *p* = 0.075, partial η^2^ = 0.0037, nor of level of education, *F*(1, 864) = 1.51, *p* = 0.220, partial η^2^ = 0.0017, on the compensation dimension.

Participants tended to report that their choices were not influenced by how many calories they thought were in menu options (kcal influence dimension). There was no significant effect of availability, *F*(1, 864) = 1.20, *p* = 0.273, partial η^2^ = 0.0014, and labelling, *F*(1, 864) = 2.86, *p* = 0.091, partial η^2^ = 0.0033, nor of level of education, *F*(1, 864) = 2.26, *p* = 0.133, partial η^2^ = 0.0026, on the *kcal influence* dimension.

- 1. *Study 2 fast food environment questionnaire analysis*

Participants tended to rate the virtual fast-food environment highly on the validity dimension. A validity score was calculated as the mean of the five items in this dimension (McDonald’s ω = 0.763). There was no significant effect of availability, *F*(1, 871) = 0.45, *p* =0.501, partial η^2^ = 0.0005, and labelling, *F*(1, 871) = 0.97, *p* = 0.325, partial η^2^ = 0.0011, but a significant effect of level of education, *F*(1, 871) = 9.26, *p* = 0.002, partial η^2^ = 0.0105, on the *validity* dimension. Participants with a higher level of education rated the virtual fast-food environment lower on the validity dimension compared to participants with a lower level of education.

Participants tended to disagree that they would have required more food after consuming their selected meal (compensation dimension). There was no significant effect of availability, *F*(1, 871) = 3.10, *p* = 0.078, partial η^2^ = 0.0036, and labelling, *F*(1, 871) = 2.23, *p* = 0.135, partial η^2^ = 0.0026, nor of level of education, *F*(1, 864) = 1.29, *p* = 0.257, partial η^2^ = 0.0015, on the *compensation* dimension.

Participants tended to report that their choices were not influenced by how many calories they thought were in menu options (kcal influence dimension). There was no significant effect of availability, *F*(1, 871) = 1.84, *p* = 0.175, partial η^2^ = 0.0021, and labelling, *F*(1, 871) = 2.33, *p* = 0.127, partial η^2^ = 0.0027, but a significant effect of level of education, *F*(1, 871) = 14.97, *p* < 0.001, partial η^2^ = 0.0169, on the *kcal influence* dimension. Participants with a higher level of education reported being more influenced by energy content than participants with a lower level of education.

1. **Detailed participants’ characteristics overall and by intervention condition**

**Table S4.** Study 1 detailed participants’ characteristics overall and by intervention condition

|  | **All**  **(n=868)** | **Baseline availability &**  **No labelling**  **(n=227)** | **Baseline availability &**  **Labelling**  **(n=218)** | **Increased availability &**  **No labelling**  **(n=194)** | **Increased availability &**  **Labelling**  **(n=229)** |
| --- | --- | --- | --- | --- | --- |
| **Age, years, mean (SD)** | 35.5 (13.4) | 35.3 (12.5) | 36.1 (14.1) | 34.8 (13.4) | 35.7 (13.6) |
| **Gender, female, n (%)** | 419 (48.27) | 111 (48.90) | 94 (43.12) | 101 (52.06) | 113 (49.34) |
| **Ethnicity, n (%)**  *White*  *Black*  *Asian*  *Mixed*  *Other* | 789 (90.90)  25 (2.88)  26 (3.00)  25 (2.88)  3 (0.35) | 204 (89.87)  6 (2.64)  9 (3.96)  7 (3.08)  1 (0.44) | 201 (92.20)  6 (2.75)  2 (0.92)  9 (4.13)  0 (0) | 174 (89.69)  6 (3.09)  7 (3.61)  5 (2.58)  2 (1.03) | 210 (91.70)  7 (3.06)  8 (3.49)  4 (1.75)  0 (0) |
| **BMI, kg/m^2^, mean (SD)**  *Missing, implausible^a^, n (%)* | 26.5 (5.78)  16 (1.84) | 26.4 (4.95)  6 (2.64) | 26.7 (6.24)  4 (1.83) | 26.3 (5.89)  3 (1.55) | 26.5 (6.02)  3 (1.31) |
| **Highest educational level, n (%)**  *No qualification*  *1–3 GCSEs*  *4+ GCSEs*  *A level*  *CertHE*  *DipHE*  *Bachelor*  *Master*  *Doctorate* | 17 (1.96)  62 (7.14)  144 (16.59)  243 (28.00)  50 (5.76)  66 (7.60)  195 (22.47)  71 (8.18)  20 (2.30) | 3 (1.32)  9 (3.96)  47 (20.70)  63 (27.75)  10 (4.41)  16 (7.05)  55 (24.23)  16 (7.05)  8 (3.52) | 4 (1.83)  22 (10.09)  33 (15.14)  62 (28.44)  11 (5.05)  22 (10.09)  37 (16.97)  19 (8.72)  8 (3.67) | 4 (2.06)  15 (7.73)  27 (13.92)  52 (26.80)  13 (6.70)  16 (8.25)  45 (23.20)  19 (9.79)  3 (1.55) | 6 (2.62)  16 (6.99)  37 (16.16)  66 (28.82)  16 (6.99)  12 (5.24)  58 (25.33)  17 (7.42)  1 (0.44) |
| **Highest educational level binary, n (%)**  Lower (≤ A Level)  Higher (> A Level) | 466 (53.69)  402 (46.31) | 122 (53.74)  105 (46.26) | 121 (55.50)  97 (44.50) | 98 (50.52)  96 (49.48) | 125 (54.59)  104 (45.41) |
| **Years of higher education, mean (SD)** | 3.17 (2.63) | 3.17 (2.61) | 3.23 (3.11) | 3.18 (2.45) | 3.10 (2.28) |
| **Equivalised income, £, mean (SD)** | 19652 (26561) | 19239 (16553) | 18168 (13559) | 19814 (39114) | 21337 (30592) |
| **Subjective social status, mean (SD)** | 4.99 (1.62) | 4.95 (1.62) | 4.96 (1.66) | 5.12 (1.59) | 4.94 (1.62) |
| **Student, yes, n (%)** | 217 (25.00) | 52 (22.91) | 57 (26.15) | 55 (28.35) | 53 (23.14) |
| **Fast-food consumption frequency, n (%)**  Never or not in the last year  Less than once per month  1-3 times per month  1-2 times per week  3 times per week or more | 17 (1.96)  242 (27.88)  436 (50.23)  155 (17.86)  18 (2.07) | 3 (1.32)  66 (29.07)  116 (51.10)  37 (16.30)  5 (2.20) | 4 (1.83)  69 (31.65)  101 (46.33)  39 (17.89)  5 (2.29) | 5 (2.58)  56 (28.87)  91 (46.91)  37 (19.07)  5 (2.58) | 5 (2.18)  51 (22.27)  128 (55.90)  42 (18.34)  3 (1.31) |
| **Dieting status, yes, n (%)** | 119 (13.71) | 39 (17.18) | 29 (13.30) | 23 (11.86) | 28 (12.23) |

^a^BMI implausible values: BMI>10 or BMI<60

**Table S5.** Study 2 detailed participants’ characteristics overall and by intervention condition

|  | **All**  **(n=875)** | **Baseline availability &**  **No labelling**  **(n=216)** | **Baseline availability &**  **Labelling**  **(n=232)** | **Increased availability &**  **No labelling**  **(n=212)** | **Increased availability &**  **Labelling**  **(n=215)** |
| --- | --- | --- | --- | --- | --- |
| **Age, years, mean (SD)** | 36.1 (12.0) | 35.2 (12.1) | 36.3 (12.3) | 36.7 (12.4) | 36.1 (11.34) |
| **Gender, female, n (%)** | 463 (52.91) | 109 (50.46) | 131 (56.47) | 114 (53.77) | 109 (50.70) |
| **Ethnicity, n (%)**  *White*  *Black*  *Asian*  *Mixed*  *Other* | 801 (91.54)  21 (2.40)  24 (2.74)  22 (2.51)  7 (0.80) | 201 (93.06)  8 (3.70)  4 (1.85)  3 (1.39)  0 (0.00) | 213 (91.81)  6 (2.59)  4 (1.72)  6 (2.59)  3 (1.29) | 192 (90.57)  5 (2.36)  7 (3.30)  6 (2.83)  2 (0.94) | 195 (90.70)  2 (0.93)  9 (4.19)  7 (3.26)  2 (0.93) |
| **BMI, kg/m^2^, mean (SD)**  *Missing, implausible^a^, n (%)* | 27.1 (5.98)  16 (1.83) | 26.3 (5.28)  5 (2.31) | 27.5 (6.15)  6 (2.59) | 27.1 (6.38)  4 (1.89) | 27.7 (6.00)  1 (0.45) |
| **Highest educational level, n (%)**  *No qualification*  *1–3 GCSEs*  *4+ GCSEs*  *A level*  *CertHE*  *DipHE*  *Bachelor*  *Master*  *Doctorate* | 15 (1.71)  52 (5.94)  119 (13.60)  286 (32.69)  62 (7.09)  73 (8.34)  195 (22.29)  63 (7.20)  10 (1.14) | 3 (1.39)  7 (3.24)  42 (19.44)  76 (35.19)  10 (4.63)  15 (6.94)  50 (23.15)  11 (5.09)  2 (0.93) | 2 (0.86)  15 (6.47)  28 (12.07)  75 (32.33)  22 (9.48)  20 (8.62)  54 (23.28)  16 (6.90)  0 (0.00) | 4 (1.89)  14 (6.60)  27 (12.74)  61 (28.77)  12 (5.66)  22 (10.38)  50 (23.58)  18 (8.49)  4 (1.89) | 6 (2.79)  16 (7.44)  22 (10.23)  74 (34.42)  18 (8.37)  16 (7.44)  41 (19.07)  18 (8.37)  4 (1.86) |
| **Highest educational level binary, n (%)**  Lower (≤ A Level)  Higher (> A Level) | 472 (53.94)  403 (46.06) | 128 (59.26)  88 (40.74) | 120 (51.72)  112 (48.28) | 106 (50.00)  106 (50.00) | 118 (54.88)  97 (45.12) |
| **Years of higher education, mean (SD)** | 3.16 (2.52) | 3.04 (2.41) | 3.19 (2.34) | 3.22 (2.51) | 3.20 (2.83) |
| **Equivalised income, £, mean (SD)** | 20296 (15139) | 19741 (14613) | 21039 (16964) | 19532 (12225) | 20805 (16178) |
| **Subjective social status, mean (SD)** | 4.95 (1.53) | 4.97 (1.60) | 5.06 (1.48) | 4.88 (1.50) | 4.87 (1.55) |
| **Employment status, n (%)**  *Full or part-time^b^*  *Student*  *Retired*  *Sick or disabled*  *Looking after home/family*  *Other unemployed* | 646 (73.83)  32 (3.66)  30 (3.43)  31 (3.54)  71 (8.11)  65 (7.43) | 162 (75.00)  10 (4.63)  7 (3.24)  7 (3.24)  15 (6.94)  15 (6.94) | 170 (73.28)  13 (5.60)  11 (4.74)  8 (3.45)  17 (7.33)  13 (5.60) | 155 (73.11)  7 (3.30)  5 (2.36)  9 (4.25)  17 (8.02)  19 (8.96) | 159 (73.95)  2 (0.93)  7 (3.26)  7 (3.26)  22 (10.23)  18 (8.37) |
| **Fast-food consumption frequency, n (%)**  Never or not in the last year  Less than once per month  1-3 times per month  1-2 times per week  3 times per week or more | 14 (1.60)  233 (26.63)  456 (52.46)  143 (16.34)  26 (2.97) | 4 (1.85)  63 (29.17)  107 (49.54)  36 (16.67)  6 (2.78) | 4 (1.72)  54 (23.28)  127 (54.74)  39 (16.81)  8 (3.45) | 2 (0.94)  57 (26.89)  111 (52.36)  37 (17.45)  5 (2.36) | 4 (1.86)  59 (27.44)  114 (53.02)  31 (14.42)  7 (3.26) |
| **Dieting status, yes, n (%)** | 121 (13.83) | 27 (12.50) | 25 (10.78) | 38 (17.92) | 31 (14.42) |

^a^BMI implausible values: BMI>10 or BMI<60. ^b^Job titles were collected but not analysed as part of this study.

1. **Sensitivity, secondary and additional analyses**

**Table S6.** ANCOVA models for sensitivity, secondary and additional analyses for Study 1, dependent variable: total energy ordered (n=868 unless otherwise specified)

| **Sensitivity analyses** | | | |
| --- | --- | --- | --- |
| **Model** | ***F*** | ***p*** | **partial η^2^** |
| **Excluding aim guessers (n=863)**  availability  labelling  level of education  availability*level of education  labelling*level of education | 28.40  0.44  0.66  2.25  0.49 | < 0.001  0.507  0.418  0.134  0.485 | 0.0321  0.0005  0.0008  0.0026  0.0006 |
| **Moderator = years in higher education**  availability  labelling  years in higher education  availability*years in higher education  labelling*years in higher education | 5.13  0.40  1.08  1.98  0.05 | 0.024  0.529  0.298  0.160  0.816 | 0.0059  0.0005  0.0013  0.0023  0.0001 |
| **Moderator = highest educational level (binary)**  availability  labelling  highest educational level  availability*highest educational level  labelling*highest educational level | 29.07  0.30  0.36  0.64  3.26 | < 0.001  0.582  0.551  0.425  0.071 | 0.0326  0.0004  0.0004  0.0007  0.0038 |
| **Secondary analyses** | | | |
| **Moderator = equivalised income**  availability  labelling  equivalised income  availability* equivalised income  labelling* equivalised income | 10.79  0.47  0.12  0.67  0.07 | 0.001  0.493  0.726  0.412  0.798 | 0.0124  0.0005  0.0001  0.0008  0.0001 |
| **Moderator = subjective social status**  availability  labelling  SSS  availability*SSS  labelling*SSS | 2.47  0.29  2.57  < 0.01  0.11 | 0.117  0.592  0.109  0.944  0.745 | 0.0029  0.0003  0.0030  < 0.0001  0.0001 |
| **Moderator = fast-food consumption frequency**  availability  labelling  fast food frequency  availability*fast-food frequency  labelling*fast-food frequency | 9.67  < 0.01  33.27  2.93  0.04 | 0.002  0.990  < 0.001  0.087  0.845 | 0.0111  < 0.0001  0.0372  0.0034  < 0.0001 |
| **Additional analyses** | | | |
| **Including available data from participants who dropped out (n=1158)**  availability  labelling  level of education  availability*level of education  labelling*level of education | 45.65  0.05  0.02  1.24  0.07 | < 0.001  0.829  0.884  0.266  0.786 | 0.0381  < 0.0001  < 0.0001  0.0011  0.0001 |

**Table S7.** ANCOVA models for sensitivity, secondary and additional analyses for Study 2, dependent variable: total energy ordered (n=875 unless otherwise specified)

| **Sensitivity analyses** | | | |
| --- | --- | --- | --- |
| **Model** | ***F*** | ***p*** | **partial η^2^** |
| **Excluding aim guessers (n=873)**  availability  labelling  level of education  availability*level of education  labelling*level of education | 16.18  1.85  2.59  0.02  0.10 | < 0.001  0.174  0.108  0.877  0.750 | 0.0183  0.0021  0.0030  < 0.0001  0.0001 |
| **Moderator = years in higher education**  availability  labelling  years in higher education  availability*years in higher education  labelling*years in higher education | 7.22  0.21  0.75  0.05  0.29 | 0.007  0.650  0.386  0.830  0.589 | 0.0082  0.0002  0.0009  0.0001  0.0003 |
| **Moderator = highest educational level (binary)**  availability  labelling  highest educational level  availability*highest educational level  labelling*highest educational level | 15.59  1.84  6.18  0.03  0.07 | < 0.001  0.176  0.013  0.871  0.785 | 0.0176  0.0021  0.0071  < 0.0001  0.0001 |
| **Secondary analyses** | | | |
| **Moderator = equivalised income**  availability  labelling  equivalised income  availability* equivalised income  labelling* equivalised income | 10.93  0.16  0.65  1.21  0.26 | 0.001  0.686  0.420  0.271  0.609 | 0.0124  0.0002  0.0008  0.0014  0.0003 |
| **Moderator = subjective social status**  availability  labelling  SSS  availability*SSS  labelling*SSS | 1.94  3.45  1.90  0.03  5.67 | 0.164  0.064  0.169  0.867  0.017 | 0.0022  0.0040  0.0022  < 0.0001  0.0065 |
| **Moderator = fast-food consumption frequency**  availability  labelling  fast food frequency  availability*fast-food frequency  labelling*fast-food frequency | 3.45  4.94  26.11  0.73  3.62 | 0.064  0.027  < 0.001  0.392  0.058 | 0.0040  0.0057  0.0324  0.0008  0.0041 |
| **Additional analyses** | | | |
| **Including available data from participants who dropped out (n=1056)**  availability  labelling  level of education  availability*level of education  labelling*level of education | 14.44  2.28  2.32  0.17  0.06 | < 0.001  0.131  0.128  0.684  0.806 | 0.0136  0.0022  0.0022  0.0002  0.0001 |

1. **Pooled analyses**
   1. *Effect of the interventions and level of education on total energy ordered*

Consistent with results of Study 1 and Study 2, pooled analyses confirmed a significant effect of availability, but no significant effect of labelling, level of education or interactions between interventions and SEP on total energy ordered (**Table S8**).

**Table S8.** Statistical models for pooled analyses, dependent variable: total energy ordered (n=1,743)

| **Additional analyses** | | | |
| --- | --- | --- | --- |
| **Model** | ***F*** | ***p*** | **partial η^2^** |
| **ANOVA**  **Moderator = highest educational level (binary)**  availability  labelling  highest educational level  availability*highest educational level  labelling*highest educational level  study | 42.49  2.13  2.07  0.14  1.91  6.72 | < 0.001  0.145  0.151  0.708  0.167  0.010 | 0.0239  0.0012  0.0012  0.0001  0.0011  0.0039 |

- 1. *Bayesian pooled analyses*

Bayes factors (denoted as ‘BF_10_’ and ‘BF_01_’) quantify evidence for each alternative model over a reference model – which can be the null model or another model chosen as reference. BF_10_=x means that the alternative model is x times more plausible than the reference model whereas BF_01_=1/BF_10_=y means that the reference model is y times more plausible than the alternative model. Bayes factors are continuous, however discrete categories have been suggested to aid interpretation. According to Raftery’s classification, a Bayes factor of 1-3 indicates a ‘weak support’, 3-20 a ‘positive support’, 20-150 a ‘strong support’, and >150 a ‘very strong support’. Bayes factors were calculated using JASP 0.9.2 and using a default prior r scale of 0.707.

**Table S9.** Bayes factors for comparison of the likelihood of total energy data under several models

| Models | BF_10_ | BF_01_ | Raftery’s classification [2] |
| --- | --- | --- | --- |
| **Reference (H0):** Null model  **Alternatives (H1):**  + availability  + labelling  + highest educational level (binary) | 8.329e+7  0.149  0.122 | 1.201e-8  6.711  8.197 | Very strong support for H1  Positive support for H0  Positive support for H0 |
| **Reference (H0):** availability + labelling + highest educational level (binary)  **Alternatives (H1):**  + availability*highest educational level (binary)  + labelling*highest educational level (binary) | 0.072  0.157 | 13.889  6.369 | Positive support for H0  Positive support for H0 |

- 1. *Moderation of interventions by participant individual differences*

We found no evidence that executive function, self-control, healthiness or weight control motivation, fast-food consumption frequency moderated the effect of availability or labelling on total energy ordered. Working memory was not associated with total energy ordered. However, participants with lower inhibitory control, higher self-control, higher healthiness motivation or higher weight control motivation ordered significantly less energy than participants with higher inhibitory control, lower self-control, lower healthiness motivation or lower weight control motivation. Participants who consumed fast-food more frequently ordered significantly more energy than participants who consumed fast-food less frequently. **See Table S10.**

**Table S10.** ANCOVA models^a^ testing the effect of the interventions and potential moderators (continuous variables) on total energy ordered (n=1,743)

|  | ***F*** | ***p*** | **partial η^2^** |
| --- | --- | --- | --- |
| **Moderator = Interference Stroop effect** |  |  |  |
| availability | 28.18 | < 0.001 | 0.0160 |
| labelling | 1.43 | 0.232 | 0.0008 |
| moderator | 23.36 | < 0.001 | 0.0133 |
| availability*moderator | 0.47 | 0.494 | 0.0003 |
| labelling*moderator | 0.01 | 0.920 | < 0.0001 |
| study | 6.67 | 0.010 | 0.0038 |
| **Moderator = Proportion of correct responses in incongruent trials from the Stroop task** |  |  |  |
| availability | 0.22 | 0.641 | 0.0001 |
| labelling | 0.31 | 0.580 | 0.0002 |
| moderator | 7.71 | 0.006 | 0.0044 |
| availability*moderator | 0.16 | 0.686 | 0.0001 |
| labelling*moderator | 0.58 | 0.448 | 0.0003 |
| study | 6.99 | 0.008 | 0.0040 |
| **Moderator = Two-error maximum length from the digit-span task** |  |  |  |
| availability | 2.83 | 0.093 | 0.0016 |
| labelling | 0.54 | 0.461 | 0.0003 |
| moderator | 0.20 | 0.656 | 0.0001 |
| availability*moderator | 0.08 | 0.781 | < 0.0001 |
| labelling*moderator | 0.09 | 0.767 | 0.0001 |
| study | 6.73 | 0.010 | 0.0039 |
| **Moderator = Maximum length from the digit-span task** |  |  |  |
| availability | 1.07 | 0.302 | 0.0006 |
| labelling | < 0.01 | 0.974 | < 0.0001 |
| moderator | 0.16 | 0.693 | 0.0001 |
| availability*moderator | 0.36 | 0.548 | 0.0002 |
| labelling*moderator | 0.18 | 0.671 | 0.0001 |
| study | 6.73 | 0.010 | 0.0039 |
| **Moderator = Self-reported self-control score** |  |  |  |
| availability | < 0.01 | 0.971 | < 0.0001 |
| labelling | 0.01 | 0.912 | < 0.0001 |
| moderator | 48.20 | < 0.001 | 0.0270 |
| availability*moderator | 2.41 | 0.121 | 0.0014 |
| labelling*moderator | 0.28 | 0.600 | 0.0002 |
| study | 7.86 | 0.005 | 0.0045 |
| **Moderator = Healthiness motivation** |  |  |  |
| availability | 1.35 | 0.246 | 0.0008 |
| labelling | 1.46 | 0.227 | 0.0008 |
| moderator | 109.40 | < 0.001 | 0.0593 |
| availability*moderator | 1.08 | 0.298 | 0.0006 |
| labelling*moderator | 0.48 | 0.487 | 0.0003 |
| study | 7.15 | 0.008 | 0.0041 |
| **Moderator = Weight control motivation** |  |  |  |
| availability | 3.04 | 0.082 | 0.0017 |
| labelling | 0.19 | 0.665 | 0.0001 |
| moderator | 92.89 | < 0.001 | 0.0508 |
| availability*moderator | 0.43 | 0.512 | 0.0002 |
| labelling*moderator | 0.03 | 0.863 | < 0.0001 |
| study | 6.86 | 0.009 | 0.0039 |
| **Moderator = Fast-food consumption frequency** |  |  |  |
| availability | 12.48 | < 0.001 | 0.0071 |
| labelling | 2.61 | 0.106 | 0.0015 |
| moderator | 62.22 | < 0.001 | 0.0346 |
| availability*moderator | 3.41 | 0.065 | 0.0020 |
| labelling*moderator | 1.52 | 0.218 | 0.0009 |
| study | 6.70 | 0.010 | 0.0038 |

^a^All moderators were treated as continuous variables

- 1. *Relationships between SEP, executive function, food choice motives and energy ordered*

Split-half reliability analyses (odd-even) were run to examine internal consistency of the Stroop and digit-span tasks across the two studies (collapsed). **Table S11** reports the split-half estimates calculated using the Spearman—Brown prophecy formula. Reliability greater than .70 or .80 is acceptable for research purposes [1].

**Table S11.** Split-half estimates for the main measures of the Stroop and digit-span tasks (n=1,743)

|  | **Stroop task** | |  | **Digit span task** |
| --- | --- | --- | --- | --- |
|  | **Median RTs for congruent trials** | **Median RTs for incongruent trials** | **Proportion of correct responses in incongruent trials** | **Maximum length** |
| Split-half estimate | 0.88 | 0.90 | 0.82 | 0.79 |

Consistent with expectations, with data collapsed across both studies (controlling for study), healthiness and weight control motivations and self-reported self-control were positively correlated with level of education and negatively correlated with total energy ordered. Working memory (both digit span task measures) was positively correlated with level of education, but no significant correlation was found between inhibition (both Stroop task measures) and level of education. Inhibition (Stroop interference) was negatively correlated with total energy ordered, whereas proportion of correct responses in incongruent trials was positively correlated. No significant correlations were found between working memory and total energy (**Table S12**).

**Table S12.** Partial correlation coefficients between level of education, total energy ordered and executive function, self-control, food choice motives adjusted for study (n=1,743)

|  | **Inhibition (Stroop task)** | | **Working memory (digit span task)** | |
| --- | --- | --- | --- | --- |
|  | **Interference Stroop effect** | **Proportion of correct responses in incongruent trials** | **Two-error maximum length** | **Maximum length** |
| Level of education^a^ | *r* = -0.04 *p* = 0.139 | *r* = 0.01 *p* = 0.707 | *r* = 0.09 *p* < 0.001 | *r* = 0.09 *p* < 0.001 |
| Total energy ordered | *r =* -0.12  *p <* 0.001 | *r =* 0.07  *p =* 0.006 | *r =* -0.01  *p =* 0.664 | *r =* 0.01  *p =* 0.681 |
|  | **Self-control score** | **Healthiness motivation^b^** | **Weight control motivation^b^** |  |
| Level of education^a^ | *r =* 0.09  *p <* 0.001 | *r =* 0.19  *p <*0.001 | *r =* 0.08  *p <* 0.001 |  |
| Total energy ordered | *r =* -0.16  *p <* 0.001 | *r =* -0.24  *p <* 0.001 | *r =* -0.21  *p <* 0.001 |  |

^a^Calculated as a continuous composite score of the z-scores for highest educational level and years in higher education. ^b^Spearman’s correlations

1. **References**

[1] M. Furr, “Split-Half Reliability,” in *Encyclopedia of Research Design*, N. J. Salkind, Ed. Thousand Oaks: SAGE Publications, Inc., 2012, pp. 1411–1413.

[2] A. E. Raftery, “Bayesian Model Selection in Social Research,” in *Sociological Methodology , Vol . 25*, vol. 25, American Sociological Association, 1995, pp. 111–163.
